# Uncovering the potential of novel micromonosporae isolated from an extreme hyper-arid Atacama Desert soil

**DOI:** 10.1038/s41598-019-38789-z

**Published:** 2019-03-18

**Authors:** Lorena Carro, Jean Franco Castro, Valeria Razmilic, Imen Nouioui, Che Pan, José M. Igual, Marcel Jaspars, Michael Goodfellow, Alan T. Bull, Juan A. Asenjo, Hans-Peter Klenk

**Affiliations:** 1 Microbiology and Genetics Department, University of Salamanca, Salamanca, Spain; 2 School of Natural and Environmental Sciences, Newcastle University, Newcastle-upon Tyne, UK; 3 Centre for Biotechnology and Bioengineering (CeBiB), Department of Chemical Engineering, Biotechnology and Materials, Universidad de Chile, Beauchef 851, Santiago, Chile; 4 Marine Biodiscovery Centre, Department of Chemistry, University of Aberdeen, Scotland, UK; 5 Instituto de Recursos Naturales y Agrobiología de Salamanca, Consejo Superior de Investigaciones Científicas (IRNASA-CSIC), Salamanca, Spain; 6 Grupo de Interacción Planta-Microorganismo, Universidad de Salamanca, Unidad Asociada al CSIC, Spain; 7 School of Biosciences, University of Kent Canterbury, Canterbury, UK

## Abstract

The taxonomic status, biotechnological and ecological potential of several *Micromonospora* strains isolated from an extreme hyper arid Atacama Desert soil were determined. Initially, a polyphasic study was undertaken to clarify the taxonomic status of five micromonosporae, strains LB4, LB19, LB32^T^, LB39^T^ and LB41, isolated from an extreme hyper-arid soil collected from one of the driest regions of the Atacama Desert. All of the isolates were found to have chemotaxonomic, cultural and morphological properties consistent with their classification in the genus *Micromonospora*. Isolates LB32^T^ and LB39^T^ were distinguished from their nearest phylogenetic neighbours and proposed as new species, namely as *Micromonospora arida* sp. nov. and *Micromonospora inaquosa* sp. nov., respectively. Eluted methanol extracts of all of the isolates showed activity against a panel of bacterial and fungal indicator strains, notably against multi-drug resistant *Klebsiella pneumoniae* ATCC 700603 while isolates LB4 and LB41 showed pronounced anti-tumour activity against HepG2 cells. Draft genomes generated for the isolates revealed a rich source of novel biosynthetic gene clusters, some of which were unique to individual strains thereby opening up the prospect of selecting especially gifted micromonosporae for natural product discovery. Key stress-related genes detected in the genomes of all of the isolates provided an insight into how micromonosporae adapt to the harsh environmental conditions that prevail in extreme hyper-arid Atacama Desert soils.

## Introduction

New natural products, especially antibiotics, are needed to control the spread of multi-drug resistant (MDR) microbial pathogens, as exemplified by MDR-resistant Gram-negative bacteria that are associated with high mortality rates^1,2^. Amongst prokaryotes, filamentous bacteria in the class *Actinobacteria*^3^ of the phylum *Actinobacteria*^4^ have a unique track record as a source of novel specialised (secondary) metabolites^5,6^. Despite this, the costly, repeated rediscovery of known chemical entities from common filamentous actinobacteria contributed to the sharp decline in the search for new clinically relevant antibiotics towards the end of the last century^7,8^. However, the discovery that the genomes of filamentous actinobacteria contained many biosynthetic gene clusters (BGCs) that encode for biosynthetic pathways of known and predicted specialised metabolites sparked a renewed interest in these organisms as a source of new chemical scaffolds^9,10^. Especially “gifted” (*sensu* Baltz^11^) actinobacteria known to have large genomes (>8.0 Mb) rich in BGCs, include *Streptomyces* strains^12–14^ and representatives of historically understudied taxa, such as the genera *Amycolatopsis*^15^, *Micromonospora*^16^ and *Saccharothrix*^17^. New approaches to the selective isolation, dereplication and screening of novel filamentous actinobacteria from neglected and unusual habitats also contributed towards the revival of interest in these organisms as a source of new specialised metabolites^18,19^.

Novel filamentous actinobacteria, notably streptomycetes, isolated from arid Atacama Desert soils are a fruitful source of new specialised metabolites^19–21^ underscoring the premise that extreme environmental conditions give rise to a unique actinobacterial diversity that is the basis of a novel chemistry^20,22,23^. Complementary metagenomic surveys of Atacama Desert habitats have revealed an enormous actinobacterial diversity most of which went undetected in corresponding culture-dependent studies^24,25^. Improved selective isolation and growth procedures can be expected to address this disparity between culture-dependent and culture-independent data, as illustrated by the discovery that members of the genus *Micromonospora* can be isolated from Atacama Desert soils^26^. This is an interesting development as micromonosporae are second only to streptomycetes as a source of new specialised metabolites^27,28^.

Carro and colleagues^16^ found that the genomes of representative *Micromonospora* type strains are a source of novel BGCs, many of which are characteristic of either individual species or groups of phylogenetically related species. Comparative genomics have also revealed phylogenetically distributed patterns of new specialised metabolites amongst members of the genus *Amycolatopsis*^29^. These observations open up the prospect of prioritising representatives of novel and rare actinobacterial taxa in the search for new specialised metabolites using state-of-the-art technologies; such as genome mining procedures^9,30^, with particular emphasis on strategies designed to activate cryptic (silent) biosynthetic gene clusters^11,31^.

The present study was designed to establish the taxonomic provenance of five *Micromonospora* strains isolated from an extreme hyper-arid Atacama Desert soil with a particular focus on their biotechnological and ecological potential. The isolates are known to be genetically diverse and four of them, namely LB4, LB39^T^ and LB19 and LB32^T^, were found to be most closely related to the type strains of *Micromonospora chalcea*^32,33^, the type species of the genus, *Micromonospora chokoriensis*^34^ and *Micromonospora saelicesensis*^35^, respectively, based on 16S rRNA gene sequence similarities^26^. The isolates and their closest phylogenetic neighbours were the subject of an extensive polyphasic taxonomic study which showed that isolates LB32^T^ and LB39^T^ represent new *Micromonospora* species for which the names *Micromonospora arida* and *Micromonospora inaquosa* are proposed. Genomes generated from all of the isolates were found to harbour many new BGCs, and carried genes adapted to deal with environmental stress that reflect their ability to adapt to extreme environmental conditions that prevail in the Atacama Desert.

## Results and Discussion

### Cultural, chemotaxonomic, morphological and genomic properties of the isolates

In general, the cultural, chemotaxonomic and morphological properties of the isolates were consistent with their classification in the genus *Micromonospora*^16,28^. The isolates were Gram-stain positive, formed extensively branched, non-fragmented substrate hyphae bearing single, non-motile spores, lacked aerial hyphae, produced orange colonies which turned brown-black on spore formation, contained *meso*-A_2_pm acid and glucose, mannose and xylose in whole-organism hydrolysates, branched chain fatty acids, hydrogenated menaquinones with nine and/or ten isoprene units and polar lipid patterns containing diphosphatidylglycerol, phosphatidylethanolamine (diagnostic lipid) and phosphatidylinositol (phospholipid type 2 *sensu* Lechevalier *et al*.^36^). The RAPD’s profiles of the isolates (Fig. S1) underpinned their genetic diversity; though isolates LB4 and LB41 gave similar profiles.

The draft genomes of isolates LB4, LB19, LB32^T^, LB39^T^ and LB41 have been deposited in GenBank under accession numbers QGSX00000000, QDGB00000000, QGSY00000000, QGSZ00000000, QGTA00000000, respectively, and are publically available. Key characteristics of the genomes are shown in Table 1; the number of contigs ranges from 339 to 1725, and the number of genes from 4976 in isolate LB4 to 7013 in LB39^T^. RNA genes represented 1–2% of the whole genome sequences ranging from 56 genes in isolate LB32^T^ to 68 in isolate LB19. The *in silico* DNA G + C contents of the genomes fell within a narrow range, namely 70.6 to 72.9%, as was the case with strains in an earlier study^16^. Isolates LB32^T^ and LB39^T^ presented similar values with 71.0 and 70.6%, while their closest type strains show values of 71.5 and 71.2 for *M*. *chokoriensis* and *M*. *saelicesensis*, respectively; results that shown coherence with values established for group IVa strains in the micromonosporal phylogenomic tree presented by Carro *et al*.^16^.

**Table 1:** Genome characteristics of the isolates.

	Isolates				
	LB4	LB19	LB32^T^	LB39^T^	LB41
Genome size	5562359	7272101	7149998	7748704	6771706
Coding sequences	4976	6678	6560	7013	6031
Number of RNAs	60	68	56	58	59
Number of contigs	1725	442	345	408	339
DNA GC content	71.8	70.9	71.0	70.6	72.9
Number of bioclusters	28	64	54	64	63

**Figure 1:**
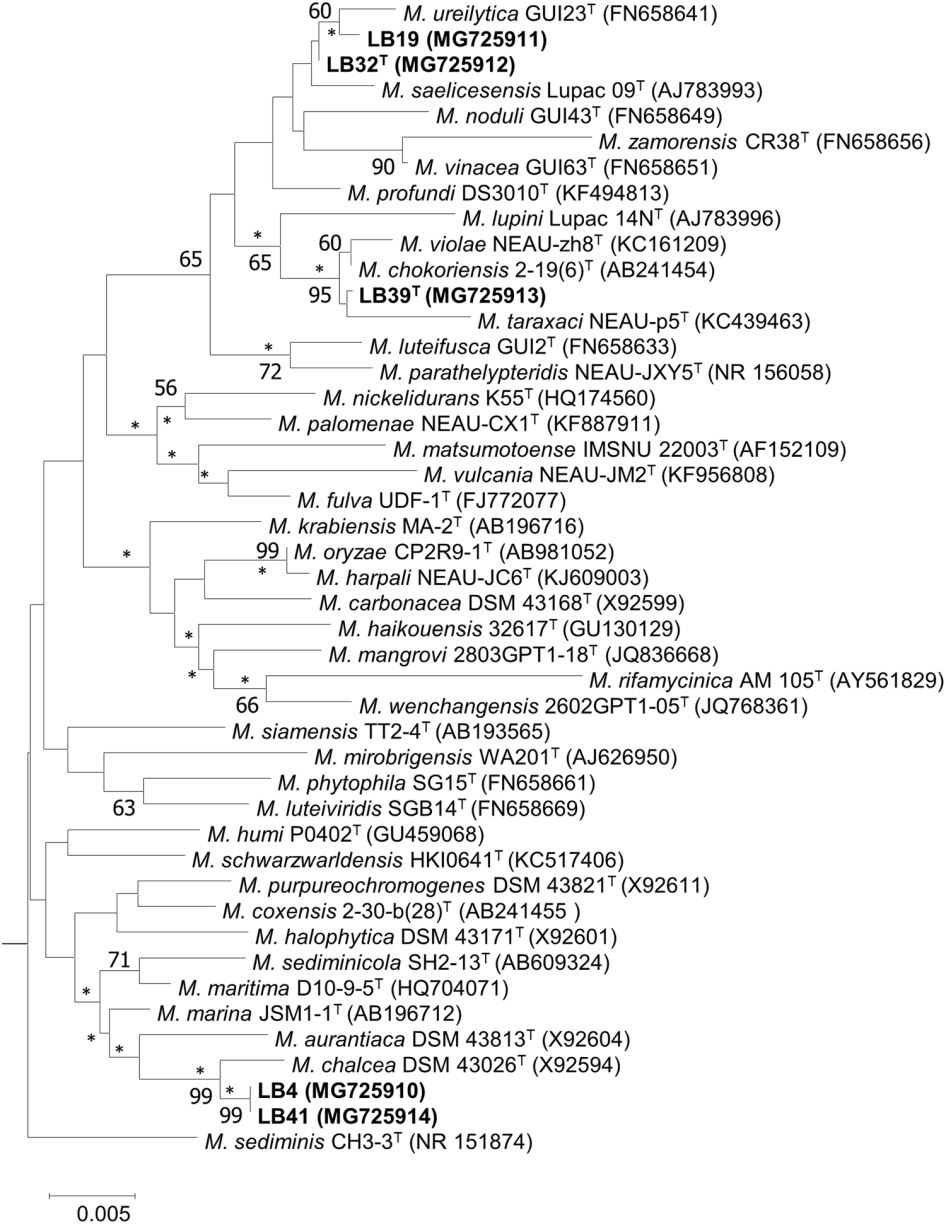
Neighbour-joining phylogenetic tree based on 16S rRNA gene sequences showing relationships between the isolates and between them and closely related *Micromonospora* type strains. The numbers at the nodes indicate bootstrap values ≥50%. Asterisks indicate branches of the tree that were also recovered in the maximum-likelihood tree. Bar, 0.005 substitutions per nucleotide position.

The positions of the isolates in the *Micromonospora* 16S rRNA gene tree are shown in Fig. S2 and their relationships with their closest phylogenetic neighbours in Fig. 1. The close relationships found between isolate LB4 and *M*. *chalcea* DSM 43026^T^, between isolate LB39^T^ and the type strains of *M*. *chokoriensis* and *Micromonospora violae*^37^ and between isolates LB19 and LB32^T^ and the type strains of *M*. *saelicesensis* and *Micromonospora ureilytica*^38^ are in good agreement with those reported by Carro *et al*.^26^. The remaining strain, isolate LB41, which was not included in the earlier analysis, was found to have an identical 16S rRNA gene sequence to isolate LB4; each of these isolates showed a corresponding sequence similarity with the type strain of *M*. *chalcea* of 99.6%. The taxonomic integrity of the *M*. *chalcea* clade is supported by a 99% bootstrap value and by the results from the maximum-likelihood and neighbour-joining analyses (Figs 1 and S1).

**Figure 2:**
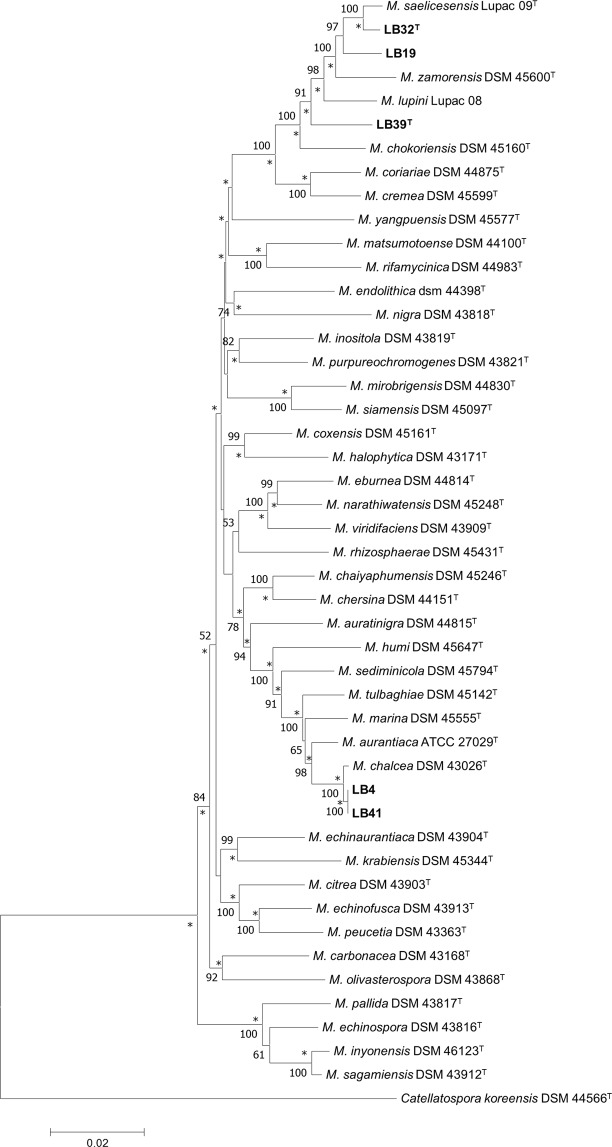
Neighbour-joining phylogenetic tree based on multilocus sequence alignment of 16S rRNA, *gyr*B, *rpo*B, *atp*D, and *rec*A gene sequences showing relationships between the isolates and between them and *Micromonospora* type strains. The numbers at the nodes are bootstrap support values when ≥50%. Asterisks indicate branches of the tree that were also recovered in the maximum-likelihood tree. *Catellatospora koreensis* DSM 44566^T^ was used as the outgroup. Bar, 0.02 substitutions per nucleotide position.

The isolates were recovered in two well supported clades based on the concatenated sequences of four housekeeping genes (*atp*D, *gyr*B, *rec*A and *rpo*B) and corresponding 16S rRNA gene sequences (Fig. 2). Isolates LB4 and LB41 belong to a clade that encompasses the type strains of *Micromonospora aurantiaca*^39^, *Micromonospora auratinigra*^40^, *Micromonospora chaiyaphumensis*^41^, *M*. *chalcea*, *Micromonospora chersina*^42^, *Micromonospora humi*^43^, *Micromonospora marina*^44^, *Micromonospora sediminicola*^45^ and *Micromonospora tulbaghia*^46^; all of these validly named species were recovered in group 1a in the *Micromonospora* phylogenomic tree generated by Carro *et al*.^16^. Isolates LB4 and LB41 were found to have identical concatenated gene sequences and showed an MLSA genetic distance with *M*. *chalcea* DSM 43026^T^ of 0.002% (Table S1), a value well below the species level threshold of ≤0.007 proposed by Rong and Huang^47,48^ and equivalent to the 70% DNA:DNA cut-off point recommended for the delineation of prokaryotic species^49^. In contrast, the two isolates shared genetic distances above the recommended threshold with all of the other closely related phylogenetic neighbours.

Isolates LB19, LB32^T^ and LB39^T^ formed a well delineated clade in the MLSA tree together with the type strains of *M*. *chokoriensis*, *Micromonospora coriariae*^50^, *Micromonospora cremea*^51^, *Micromonospora lupini*, *M*. *saelicesensis*^35^ and *M*. *zamorensis*^51^; all of these validly named species were recovered in group IVa in the phylogenomic tree of Carro *et al*.^16^. The type strains of *Micromonospora noduli*, *Micromonospora ureilytica* and *Micromonospora vinacea*^38^ can be added to this group as they have been shown to be closely related both to one another and to the *M*. *saelicesensis* and *M*. *zamorensis* strains in phylogenetic tress based on *gyr*B, MLSA, and 16S rRNA gene sequences^52,53^.

Isolate LB32^T^ formed a well-supported lineage in the MLSA tree together with *M*. *saelicesensis* Lupac09^T^; isolate LB19 was found at the periphery of this taxon, albeit as a distinct branch (Fig. 2). It is apparent from the MLSA distance score of 0.008 that isolate LB32^T^ and *M*. *saelicesensis* are distinct, but sister, species (Table S1). In contrast, it is clear that isolate LB19 belongs to the species *M*. *ureilytica* as the two strains share a distance score of 0.005, well below the cut-off point for assigning strains to the same species^47,48^. On the same basis, it is evident from Table S1 that isolate LB39^T^ shows distance scores with its nearest relatives above the 0.007 threshold and thereby merits consideration as a new *Micromonospora* species.

The isolates can be distinguished from one another by a broad range of phenotypic properties providing further evidence that they are not clones (Table 2). Excellent congruence was found between the standard phenotypic tests carried out in duplicate though this was not the case with some of the Biolog tests, many of which were weakly positive. The results of all of the phenotypic tests carried out on the isolates can be compared with those of the reference strains as the latter were recorded using the same media and methods. In general, all of the strains grew well from 20–37 °C, at pH 7 and 8, in the presence of 1% w/v sodium chloride, were catalase positive, active in the API-ZYM tests and oxidised a broad range of carbon compounds.

**Table 2:** Phenotypic properties distinguishing the isolates from one another and from their closest phylogenetic neighbours.

	Isolate LB4	Isolate LB41	*M*. *chalcea* DSM 43026^T^	Isolate LB19	*M*. *ureilytica* GUI23^T*^	Isolate LB32^T^	*M*. *saelicesensis* Lupac 09^T^	Isolate LB39^T^	*M*. *chokoriensis* JCM 13248^T^
**Biochemical tests:**									
Catalase	**+**	**+**	−	**+**	**+**	**+**	**+**	**+**	**+**
Oxidase	−	−	−	−	**+**	−	**+**	**+**	**+**
**API ZYM tests:**									
Acid phosphatase	+	+	+	+	−	−	+	+	+
Alkaline phosphatase	+	+	+	−	+	−	−	+	+
α-Chymotrypsin	+	+	+	+	+	−	+	+	−
Cystine arylamidase	+	+	−	+	+	+	+	+	+
α-Galactosidase	+	+	+	+	−	+	+	+	+
N-acetyl-β-Glucosaminidase	−	−	+	+	+	+	+	+	−
α-Glucosidase	+	+	+	+	−	+	+	+	+
β-Glucosidase	+	+	+	+	−	+	+	+	+
β-Glucuronidase	+	+	+	+	−	+	−	+	+
α-Fucosidase	−	−	−	−	−	−	+	−	−
Lipase (C14)	+	+	+	+	−	+	+	+	+
Leucine arylamidase	+	+	−	+	+	+	+	+	+
α-Mannosidase	−	−	−	+	−	−	+	+	−
Valine arylamidase	+	+	−	+	+	+	+	+	+
**GENIII BIOLOG microplate tests:**									
**(a) Oxidation of amino acids:**									
L-Alanine	+	−	−	−	−	+	+	+	+
L-Arginine	+	+	+	−	−	+	−	+	−
D-Aspartic acid	+	+	+	−	−	−	−	−	−
Glycyl-L-proline	−	−	−	−	−	−	+	−	+
L-Histidine	−	−	+	−	−	−	−	−	−
D-Serine #2	−	−	−	−	−	−	−	+	−
L-Serine	−	−	−	−	−	+	−	+	+
**(b) Oxidation of sugars:**									
D-Arabitol	−	−	−	+	+	−	−	−	−
D-Fucose	−	−	+	−	−	−	−	−	−
L-Fucose	−	+	+	+	+	+	+	+	+
3-O-methyl-D-Glucose	−	+	+	+	+	+	+	+	+
N-acetyl-D-Galactosamine	+	−	+	+	+	+	+	+	+
Glucuronamide	−	−	−	−	−	−	−	−	+
Glycerol	−	+	+	−	−	+	−	+	+
*myo*-Inositol	−	−	−	−	−	+	+	−	−
α-D-Lactose	+	+	+	−	−	−	−	+	+
D-Mannitol	−	−	−	+	+	+	−	−	+
L-Rhamnose	−	−	+	−	−	+	+	+	+
D-Salicin	−	+	+	+	+	+	+	+	+
D-Sorbitol	−	−	+	+	+	+	+	−	+
**(c) Oxidation of organic acids:**									
Bromo-Succinic acid	−	−	−	+	+	+	+	+	−
Butyric acid	+	+	+	−	−	−	−	+	−
β-*hydroxy*-Butyric acid	+	+	−	+	+	+	+	−	+
γ-amino-*n*-Butyric acid	−	−	+	−	−	−	−	−	−
Citric acid	−	−	+	−	−	−	−	−	−
D-Galacturonic acid	+	−	+	+	+	+	−	+	+
β-*methyl*-D-Glucoside	−	−	+	+	+	+	+	+	+
D-Glucuronic acid	+	−	−	+	+	+	−	+	+
α-*keto*-Glutaric acid	+	−	+	+	+	+	−	−	−
D-Lactic acid methyl ester	+	+	-	+	+	+	+	+	+
D-Malic acid	−	+	+	−	−	−	−	−	+
L-Malic acid	+	+	−	+	+	+	+	+	+
N-acetyl-Neuraminic acid	+	+	+	−	−	+	−	−	−
L-Pyroglutamic acid	+	+	−	−	−	+	+	−	−
Quinic acid	+	+	−	−	−	−	−	−	+
D-Saccharic acid	−	−	−	+	+	+	+	+	+
**(d) Oxidation of polymer:**									
Pectin	+	+	+	+	+	+	+	−	+
**Growth in the presence of:**									
Inosine	+	+	+	+	+	+	+	+	−
Lithium chloride	+	+	−	−	−	−	+	−	−
Minocycline	−	−	−	−	−	−	−	+	−
Potassium tellurite	−	+	−	−	−	+	+	+	−
Sodium chloride (4%, w/v)	+	+	−	−	−	−	−	−	−
Sodium bromate	+	+	−	−	−	−	+	−	−
Sodium formate	−	−	−	−	−	−	+	−	−
Sodium lactate (1%, w/v)	+	+	−	−	−	−	+	−	−
**Tolerance tests:**									
Temperature range (°C)	20–45	20–45	20–37	20–37	20–37	20–37	12–45	12–37	12–45
pH range	6–9	6–9	7–9	6–8	7–8	6–8	7–9	6–8	6–9
Growth in the presence of NaCl (%, w/v)	4	4	1	3	1	1	1	3	1
**Chemotaxonomy:**									
**Diaminopimelic acid**	***meso*** **-A**_**2**_ **pm**	***meso*** **-A**_**2**_ **pm**	***meso*** **-A**_**2**_ pm*	***meso*** **- and OH-A**_**2**_ **pm**	***meso*** **-A**_**2**_ pm*	***meso*** **- and OH-A**_**2**_ **pm**	***meso*** **-A**_**2**_ pm*	***meso*** **- and OH-A**_**2**_ **pm**	***meso*** **-A**_**2**_ pm*
Fatty acids	*iso*-C_16:0_ (31.0%), *iso*-C_15:0_ (10.6%), *iso*-C_17:0_ (11.2%), *anteiso*-C_17:0_ (15.1%)	*iso*-C_16:0_ (38.5%), *iso*-C_15:0_ (7.7%), *iso*-C_17:0_ (6.4%), *anteiso*-C_17:0_ (11.0%)	*iso*-C_16:0_, *iso*-C_15:0_, *anteiso*-C_17:0,_ *iso*-C_17:1_ω9c*	*iso*-C_16:0_ (11.0%), *iso*-C_15:0_ (27.0%), ante*iso*-C_17:0_ (10.4%)	*iso*-C_15:0_, *iso*-C_17:1_*ω*9c, *iso*-C_17:0_, *anteiso*-C_17:0_*	*iso*-C_15:0_ (23.8%), *iso*-C_17:0_ (14.6%), *anteiso*-C_17:0_ (12.1%)	*iso*-C_16:0_, *iso*-C_15:0_, C_17:1_ cis8*	*iso*-C_15:0_ (23.5%), *iso*-C_16:0_ (14.8%), 10-methyl C_17:0_ (14.0%), C_17:0_ (10.8%)	*iso*-C_16:0_, *iso*-C_15:0_, *iso*-C_17:0_, *anteiso*-C_15:0*_
Menaquinones	MK-9(H_4_,H_6_) (24.5, 17.6%) MK-10(H_4_,H_6_) (18.4, 16.6%)	MK-9 (H_4_,H_6_) (23.1, 10.2%) MK-10(H_4_,H_6_) (40.7, 17.6%)	MK-10(H_4_,H_6_)*	MK-10(H_4_,H_6_) (54.8, 18.1%)	MK-9 (H_4_) MK-10(H_4_,H_6_)*	MK-10(H_4_,H_6,_H_8_) (31.9, 28.9, 19.3%)	MK-10(H_4_,H_6_)*	MK-10(H_4_,H_6,_H_8_) (31.7, 24.3, 13.3%)	MK-9 (H_4_,H_6_) MK-10(H_4_,H_6_)*
Polar lipids	DPG, PE, PI, GL, 2PL	DPG, PE, PI, GL, 2PL	ND	DPG, PE, PI, GL, 5 L	DPG, PE, PI, GL*	DPG, PE, PI, GL, 2 L	DPG, PE, PI*	DPG, PE, PI, GL, 2PL	DPG, PE, PI, PIM*
Sugars	gal, glu, man, xyl	gal, glu, man, xyl	ND	gal, glu, man, rham, rib, xyl	gal, glu, man, rib, xyl*	gal, glu, man, rham, rib, xyl	ara, glu, man, rham, rib, xyl*	gal, glu, man, rham, rib, xyl	ara, gal, glu, man, rham, rib, xyl*

+: positive, −:negative, abreviations: dpm: diaminopimelic acid, ara: arabinose, gal: galactose, glu: glucose, man: mannose, rha: rhamnose, rib: ribose, xyl: xylose, DPG: diphosphatidylglycerol, PE: phosphatidylethanolamine, PI: phosphatidylinositol, PIM: phosphatidylinositol mannosides, GL: glycolipids, PL: unknown polar lipids, L: unknown lipids. All tests are from this study unless indicated otherwise. *Data from Genilloud^28^, Ara and Kudo^34^, Carro *et al*.^38^ and Trujillo *et al*.^35^.

All the isolates grew at pH 6 and in the presence of sodium chloride (1%, w/v) but not in the presence guanidine hydrochloride, niaproof, tetrazolium blue or tetrazolium violet. They were resistant to aztreonam, nalidixic acid, rifamycin SV, but not to fusidic acid, lincomycin, troleandomycin or vancomycin. All of the strains were positive for esterase (C4), esterase lipase (C8), β-galactosidase, naphtlol-AS-BI-phosphohydrolases and trypsin (API tests), oxidized acetic acid, acetoacetic acid, L-aspartic acid, D-gluconic acid, L-glutamic acid, and propionic acid (organic acids), D-cellobiose, D-fructose, D-fructose-6-phosphate, D-galactose, β-gentiobiose, N-acetyl-D-glucosamine, D-glucose, D-glucose-6-phosphate, D-maltose, N-acetyl-β-D-mannosamine, D-mannose, D-melibiose, D-raffinose, stachyose, sucrose, D-trehalose and D-turanose (sugars), degraded dextrin, gelatin and Tween 40. In contrast, none of the strains oxidized D-serine I (amino acid), α-*hydroxi*-butyric acid, α-*keto*-butyric acid, L-galactonic acid*-γ*-lactone, L-lactic acid, mucic acid and p-*hydroxy*-phenylacetic acid.

The close relationship recorded earlier between isolates LB4 and LB41 was underpinned by the results from the chemotaxonomic and phenotypic analyses (Table 2). The strains were found to have identical profiles for the biochemical, enzymatic and tolerance tests and showed a similar ability to oxidise organic acids and sugars. Whole organism hydrolysates of the isolates contained *meso*-A_2_pm, glucose, galactose, mannose and xylose; they were also shown to have identical polar lipid patterns. In addition, the major fatty acid of strains LB4 and LB41 was *iso*-C_16:0_ (31.0 and 38.5%, respectively) and the predominant isoprenologue MK-9 (H_4_) (24.5 and 23.1%). When compared with the profiles of the reference strains isolates LB4 and LB41 were most closely related to the type strain of *M*. *chalcea* showing overall phenotypic similarities with the latter of 76 and 77% indicating that all three strains belong to the same taxospecies^54,55^. These strains can be distinguished from all of the other organisms given their ability to metabolise D-aspartic acid and inability to oxidise D-saccharic acid. Similarly, the close relationship found earlier between isolate LB19 and the type strain of *M*. *ureilytica* is underpinned by the phenotypic data; these strains have many more unit characters in common than isolate LB19 has with the other reference type strains. Isolate LB19 and *M*. *ureilytica* GUI23^T^ can be distinguished from all of the other organisms given their ability to oxidise D-arabitol. They also share similar whole organism hydrolysate and polar lipid patterns (Table 2).

Isolate LB32^T^ can be separated readily from the type strain of *M*. *saelicesensis*, its closest phylogenetic neighbour, by a broad range of phenotypic properties, as exemplified by its ability to produce β-glucoronidase, oxidise L-serine, D-galacturonic acid, D-glucuronic acid, α-*keto*-glutaric acid, N-acetyl-neuraminic acid, glycerol and D-mannitol. In turn, *M*. *saelicesensis* strain Lupac 09^T^, unlike isolate LB32^T^, grew at pH 9.0 and 45 °C, showed much greater activity in the API-ZYM tests, was oxidase positive, oxidised glycyl-L-proline and D-sorbitol and grew in the presence of lithium chloride, sodium bromate and sodium formate. These differential characters are underscored by several chemotaxonomic traits, notably differences in fatty acid and whole cell sugar patterns (Table 2).

Isolate LB39^T^ can be distinguished from the type strain of *M*. *chokoriensis*, its closest phylogenetic neighbour, using a combination of chemotaxonomic and other phenotypic features (Table 2). The former, unlike the latter, produces α-mannosidase, oxidises L-arginine, D-serine #2, butyric acid and bromo-succinic acid and grows in the presence of minocycline, sodium chloride (4%, w/v) and potassium tellurite. In contrast, only the reference strain grows at pH 9.0 and 45 °C, degrades pectin and oxidises L-histidine, α-*hydroxy*-butyric acid, D-malic acid, D-mannitol and D-sorbitol. The two organisms can also be distinguished using key chemical markers, as illustrated by differences in menaquinone, polar lipid and whole cell sugar composition.

ANI (average nucleotide identity) and dDDH (digital DNA-DNA hybridization) values were calculated between the isolates and between them and their closest phylogenetic neighbours, as shown in Table 3. It is apparent on both counts that isolates LB4 and LB41 are *bona fide* members of the species *M*. *chalcea* as they share ANI and dDDH values with the latter well above the 99.5–99.6%^47,48^ and 70% thresholds^49^ used to assign strains to the same genomic species. It is also apparent that isolates LB19 and LB39^T^ are not closely related to one another or to any of the other strains included in these analyses. The situation with respect to isolate LB32^T^ appears to be less clear cut as it shares a dDDH value with the type strain of *M*. *saelicesensis* of 68.2% though the corresponding ANI value, 96.2%, is above the ANI threshold. Similar anomalies have been observed between other closely related *Micromonospora* species, as with the type strains of *Micromonospora carbonacea* and *Micromonospora haikouensis* which shared dDDH and OrthoANI values of 59.9 and 95.2%, respectively; while the corresponding values for the type strains of *Micromonospora inyonensis* and *Micromonospora sagamiensis* were 68.9% and 96.5%^16^. Indeed, some species which have been conclusively shown to belong to different *Micromonospora* species sport higher dDDH and ANI values, as exemplified by the type strains of *Micromonospora noduli* and *M*. *saelicesensis* which share dDDH and OrthoANI values of 71.2 and 96.8%, respectively^56^. Corresponding data between isolate LB19 and the type strain of *M*. *ureilytica* cannot be determined until the whole genome sequence of the latter becomes available though the 99.6% MLSA value found between these strains indicates that they belong to the same genomic species, namely *M*. *ureilytica*^38^.

**Table 3:** Average nucleotide indices and digital DNA:DNA hybridization values (%) between the isolates and between them and their closest phylogenetic neighbours.

	Isolate LB4	Isolate LB19	Isolate LB32^T^	Isolate LB39^T^	Isolate LB41	*M*. *aurantiaca* ATCC 27029^T^	*M*. *chalcea* DSM 43026^T^	*M*. *chokoriensis* DSM 45160^T^	*M*. *coriariae* DSM 44875^T^	*M*. *lupini* Lupac 08	*M*. *marina* DSM 45555^T^	*M*. *noduli* GUI43^T^	*M*. *saelicesensis* Lupac 09^T^	*M*. *tulbaghiae* DSM 45142^T^
Isolate LB4	—	81.9	82.1	81.9	**99**.**8**	93.4	**98**.**5**	81.8	82.6	82.2	92.5	93.3	82.2	80.8
Isolate LB19	26.8	—	93.5	92.7	80.8	80.6	80.7	89.1	87.7	87.7	80.6	93.7	93.8	80.7
Isolate LB32^T^	26.8	54.1	—	92.7	81.0	80.9	80.9	89.3	87.8	87.6	80.8	95.8	**96**.**2**	80.8
Isolate LB39^T^	27.0	50.7	50.5	—	80.8	80.6	80.7	89.0	87.8	87.2	80.6	92.8	92.9	80.6
Isolate LB41	**99**.**2**	24.9	24.8	25.0	—	93.2	**98**.**7**	80.6	81.4	81.1	92.2	80.9	81.1	92.9
*M*. *aurantiaca* ATCC 27029 ^T^	54.6	24.7	24.7	24.7	52.4	—	93.0	80.5	81.3	80.9	94.1	80.8	80.9	94.5
*M*. *chalcea* DSM 43026^T^	**89**.**2**	24.8	24.7	24.9	**89**.**7**	51.5	—	80.4	81.3	81.0	92.1	80.8	80.9	92.9
*M*. *chokoriensis* DSM 45160^T^	26.9	38.3	38.3	37.8	24.5	24.3	24.3	—	87.4	87.1	80.4	80.8	89.1	80.8
*M*. *coriariae* DSM 44875^T^	27.5	35.6	35.6	35.7	25.3	25.3	25.1	34.4	—	87.9	81.3	87.8	87.9	81.3
*M*. *lupini* Lupac 08	27.3	35.3	34.9	34.7	25.2	25.1	25.0	33.7	35.9	—	80.9	87.7	87.8	81.0
*M*. *marina* DSM 45555^T^	51.3	24.6	24.6	24.6	49.3	57.2	48.7	24.2	25.1	24.9	—	80.7	80.9	93.3
*M*. *noduli* GUI43^T^	26.8	55.3	66.1	50.4	24.7	24.6	24.6	37.9	35.6	35.0	24.5	—	**96**.**6**	80.8
*M*. *saelicesensis* Lupac 09^T^	26.9	55.6	68.4	50.7	24.7	24.5	24.5	37.9	35.7	34.9	24.5	**71**.**2**	—	80.9
*M*. *tulbaghiae* DSM 45142^T^	53.9	24.6	24.6	24.6	51.2	60.1	51.3	24.6	25.1	24.9	55.0	24.5	24.5	—

In summary, it can be concluded that isolates LB4 and LB41 exhibit a broad range of taxonomic properties consistent with their assignment to the validly named species, *M*. *chalcea*^32,33^, strains of which have been isolated from air, soil and aquatic habitats^28^. It is also evident that isolate LB19 belongs to the recently recognised species, *M*. *ureilytica*^38^, the sole representative of which came from a root nodule of *Pisum sativum*. These results provide further evidence that representatives of *Micromonospora* species are widely distributed in the environment^16^ though there is evidence that micromonosporae are a feature of extreme habitats^57–59^. It is also apparent that isolate LB32^T^, which forms a sister clade to the type strain of *M*. *saelicesensis* can be distinguished from the latter by a rich assortment of chemotaxonomic, genotypic and phenotypic data. Similarly, strain LB39^T^ merits recognition as a novel *Micromonospora* species as a wealth of taxonomic data can be weighted to separate it from the type strain of *M*. *chokoriensis*. In light of these results it is proposed that isolates LB32^T^ and LB39^T^ be recognized as new *Micromonospora* species for which we propose the names *Micromonospora arida* sp. nov. and *Micromonospora inaquosa* sp. nov., respectively.

None of the isolates inhibited the growth of the *B*. *subtilis*, *E*. *coli* and *P*. *fluorescens* strains in previous plug assays^26^, possibly due to the use of an inadequate cultivation media. In contrast, extracts from all of the isolates were shown to be active against the bacterial and fungal indicator strains, as shown in Table 4 where extracts showing the greatest activity are given in bold. In general, the most pronounced activity was seen in fractions eluting at higher concentrations of methanol, as exemplified by the inhibition of the *E*. *coli* and *K*. *pneumoniae* strains. Extracts showed relatively little activity against the *A*. *baumannii*, *A*. *fumigatus* and *P*. *fluorescens* strains and only moderate inhibition of the methicillin-resistant and methicillin-sensitive strains of *S*. *aureus*. Similarly, little activity was found against the *C*. *albicans* strain with the exception of extracts from isolate LB41. Interestingly, only extracts from isolates LB4 and LB41 showed pronounced inhibition of human hepatocellular carcinoma (HepG2) cells. These results are not only promising, but also provide further evidence that novel and rare micromonosporae from previously unexplored habitats are a promising source of antimicrobial agents^60,61^.

**Table 4:** Ability of different fractions of polar compounds extracted from the strains isolated from Lomas Bayas soil to inhibit: 1, *Acetobacter baumannii* CL5973; 2, *Escherichia coli* ATCC 25922; 3, *Klebsiella pneumoniae* ATCC 700603; 4, *Pseudomonas aeruginosa* MB5919; 5,6 methicillin resistant/sensitive *Staphilococcus aureus* MB5393; 6, *Staphilococcus aureus* ATCC 23213; 7, *Aspergilllus fumigatus* ATCC 46645; 8, human hepatocellular carcinoma (HepG2) cells. Values under 50% are marked in bold.

Strain ID	Fraction	Conc. [ug/mL]	1		2		3		4		5		6		7		8			
			% INH	SE	% INH	SE	% INH	SE	% INH	SE	% INH	SE	% INH	SE	% INH	SE	Conc. [mg/l]	Activity	SE	*p*-Value
LB4	Water	300	62.8	51.9	−4.6	12.0	−14.1	0.6	19.1	13.4	2.7	8.0	70.7	27.1	−5.8	11.6	75	−31.6	14.4	0.04
LB4	25% methanol	300	−9.1	13.2	−18.5	15.3	−21.0	8.1	−4.6	19.1	−12.0	5.1	−11.6	3.5	−5.6	4.0	75	2.4	4.3	0.53
LB4	50% methanol	300	−15.7	13.7	−21.4	5.0	−18.8	4.7	−5.3	26.2	−13.7	5.4	−20.0	2.5	−3.0	10.4	75	25.4	20.2	0.00
LB4	75% methanol	300	14.8	30.9	−20.5	1.0	−34.4	1.1	26.9	17.6	−14.0	5.5	−18.0	4.6	0.1	0.9	75	31.3	1.7	0.80
LB4	100% methanol	150	3.2	—	−36.7	3.9	−57.0	1.9	22.8	—	−17.5	8.5	−21.4	4.5	−1.9	2.3	75	−39.9	10.2	0.14
LB4	100% methanol + 0.01% TFA	150	58.0	—	−28.5	6.5	−53.9	2.3	21.5	—	−13.6	10.6	−11.8	1.7	−0.1	2.5	75	27.6	2.7	0.70
LB19	Water	300	−8.6	17.5	−4.3	6.7	3.2	30.9	−4.8	20.6	3.0	20.3	50.0	33.2	−1.4	5.3	75	10.8	13.8	0.05
LB19	25% methanol	300	−17.5	7.5	5.1	5.8	1.8	12.4	−6.2	21.4	−15.2	3.3	−10.5	1.4	−7.3	10.3	75	10.9	5.3	0.45
LB19	50% methanol	300	62.0	34.7	−9.8	0.2	−20.1	0.0	23.0	24.4	−13.9	6.1	−2.2	4.0	−3.0	7.1	75	−24.3	13.0	0.06
LB19	75% methanol	300	67.2	21.9	−18.4	1.6	−36.3	2.7	31.6	32.7	−12.6	6.4	−18.5	1.4	−4.5	3.7	75	18.6	12.8	0.07
LB19	100% methanol	300	83.4	24.4	−29.4	3.5	−46.1	5.7	39.4	42.6	−17.7	5.8	−15.9	11.1	−2.4	8.9	75	17.1	7.5	0.28
LB19	100% methanol + 0.01% TFA	150	61.6	—	−20.2	0.8	−56.5	1.9	15.6	—	−13.2	9.0	−15.4	3.8	−0.7	3.3	75	−2.1	13.2	0.06
LB32^T^	Water	300	33.2	66.0	−1.1	11.2	−15.2	5.0	−0.1	16.4	10.8	4.8	44.9	5.6	−0.5	7.2	75	−18.1	19.3	0.01
LB32^T^	25% methanol	300	−8.2	15.9	6.1	6.1	−15.6	4.5	−2.1	22.3	−16.8	5.1	−19.8	11.8	−3.0	3.0	75	42.7	1.7	0.81
LB32^T^	50% methanol	300	10.0	10.2	−11.6	0.5	−9.9	10.1	0.2	7.4	−11.4	3.9	−14.0	3.4	2.0	4.0	75	63.5	19.3	0.01
LB32^T^	75% methanol	300	−7.8	18.3	−12.7	1.8	−12.7	2.6	−3.4	22.3	−11.9	7.4	−17.4	2.0	−0.2	3.5	75	41.0	17.4	0.01
LB32^T^	100% methanol	300	83.2	20.6	−27.5	2.6	−44.3	3.4	42.1	42.3	−16.3	9.1	−22.4	4.1	−1.4	0.7	75	1.1	14.1	0.04
LB32^T^	100% methanol + 0.01% TFA	300	60.6	17.7	−29.6	0.2	−58.6	0.8	33.2	25.3	−18.2	6.0	−20.0	3.2	−6.3	1.9	75	31.2	6.2	0.37
LB39^T^	Water	300	−8.7	17.3	−9.4	3.1	−26.1	3.0	−2.9	23.2	−12.5	8.5	−12.5	2.9	1.3	3.2	75	14.3	8.9	0.20
LB39^T^	25% methanol	300	3.8	41.7	1.7	0.3	−16.6	3.8	−12.4	16.7	−16.7	5.2	−18.1	0.5	−3.4	5.3	75	35.0	0.9	0.89
LB39^T^	50% methanol	300	−6.5	15.7	−11.2	4.3	−21.9	2.8	−3.8	17.2	−13.1	6.8	−20.9	0.4	−1.7	2.6	75	25.5	20.8	0.00
LB39^T^	75% methanol	300	57.5	54.9	−27.5	0.2	−30.9	1.9	10.5	15.8	−12.8	4.2	−11.9	3.1	1.9	6.5	75	26.0	10.4	0.13
LB39^T^	100% methanol	300	75.2	26.1	−26.7	3.3	−37.8	1.6	32.5	38.7	−17.1	7.8	−18.2	0.0	−1.8	4.1	75	−5.1	0.9	0.90
LB39^T^	100% methanol + 0.01% TFA	300	51.8	29.5	−30.6	1.9	−60.5	0.8	16.3	25.4	−12.8	3.9	−15.0	1.7	−3.8	0.5	75	64.0	8.1	0.24
LB41	Water	300	−8.1	10.0	−10.5	1.8	−23.6	3.9	−4.0	13.5	−14.4	7.9	−17.7	5.2	1.7	3.5	75	−16.5	15.3	0.03
LB41	25% methanol	300	−15.9	15.0	−10.6	1.8	−29.6	3.3	−9.3	22.3	−14.8	4.5	−18.0	6.2	−0.8	5.3	75	16.8	17.2	0.01
LB41	50% methanol	300	63.6	29.1	−28.6	10.2	−9.1	2.1	24.2	27.6	−7.0	7.1	−7.3	0.6	−4.0	4.3	75	37.4	8.1	0.24
LB41	75% methanol	300	44.9	24.4	−18.5	8.6	−30.2	0.9	16.9	18.7	−11.6	4.1	−4.7	2.1	−4.5	0.5	75	37.9	12.5	0.07
LB41	100% methanol	300	61.5	27.9	−32.6	1.5	−42.3	5.9	26.2	22.1	−17.6	7.8	−19.2	1.9	−10.0	0.9	75	−49.7	6.4	0.36
LB41	100% methanol + 0.01% TFA	300	30.1	62.3	−14.4	2.1	−6.7	1.2	0.9	18.6	−10.7	8.4	−17.3	3.7	1.4	2.7	75	47.1	0.3	0.97
−	Negative Control	150	9.0	10.9	−8.0	1.1	−2.0	5.8	17.4	1.8	−8.0	1.9	−8.0	1.5	−5.0	2.8	500	4.8	14.6	—
+	Positive Control (MMS)	—	—	—	—	—	—	—	—	—	—	—	—	—	—	—	4	−99.9	0.2	—

% INH: percentage of inhibition; SE: Standard Error; TFA: trifluoroacetate; MMS: Methyl methane sulphonate.

### Genetic potential of the isolates to produce specialised metabolites

**Figure 3:**
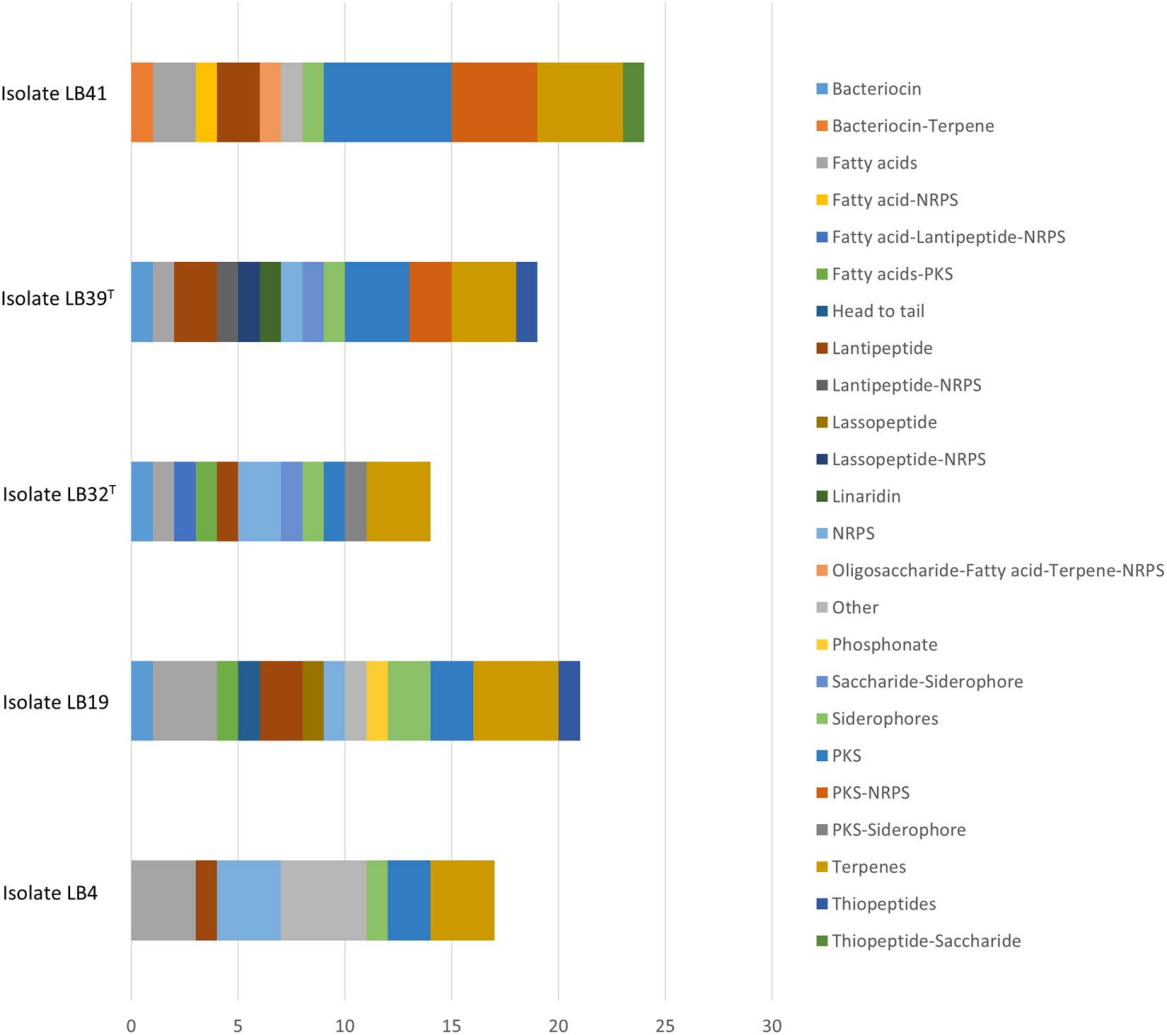
Biosynthetic gene clusters found in the genomes of isolates LB4, LB19, LB32^T^, LB39^T^, and LB41 using antiSMASH 4.0.

The draft genomes of all of the isolates were examined using the antiSMASH server to detect putative BGCs. The number of such bioclusters ranged from 28 in the genome of isolate LB4 to 64 in the genomes of isolates LB19 and LB41 though this lower number may be a function of a low quality genome, as shown by the relatively high number of contigs (Table 1). Even so, the number of BGCs found in the genomes of the isolates is well within the range found in those of the *Micromonospora* type strains examined by Carro *et al*.^16^. In contrast, the average number of BGCs detected in the genomes of the isolates, namely 54, is more than double the average number reported in the earlier study^16^. However, as in that study, the predominant BGCs types coded for lantipeptides, non-ribosomal peptide synthases, polyketide synthases, siderophores and terpenes (Table S2; Fig. 3).

The genomes of the isolates contained 25 BGCs encoding for compounds that showed some degree of similarity to specialised metabolites not previously found in *Micromonospora* strains. The genomes of all of the isolates encode for BGCs that showed a genetic correspondence to coumermycin, an amino-coumarin antibiotic, produced by *Streptomyces rishiriensis* strain DSM 40489^62^, which is known to inhibit DNA gyrase and bacterial cell division^63^. In the same vein the genomes of all of the isolates code for a BGC that shows a similarity, *ca*. ∼40%, to lymphostin biosynthetic cluster, an immunosuppressant originally found in *Streptomyces* sp. KY11783^64^. Finally, all of the isolates have the capacity to produce compounds related to diazepinomicin, a small alkaloid molecule that binds to and inhibits Ras kinase with the potential to treat multiple solid tumours^65^; this compound was first detected in the marine actinobacterium, *Micromonospora* sp. DPJ12^66^. The genome of isolate LB32^T^ contained a BGC that showed a relatively low similarity to that of algamycin I, an antibacterial 16-membered macrolide active against *Micrococcus luteus* and *Salmonella typhimurium*, a compound initially found in *Streptomyces* sp. KMA-011^67^.

It is also interesting that 18 out of these 25 BGCs were discontinuously distributed across the genomes of the isolates: 1, 5, 3, 2 and 7 in strains LB4, LB19, LB32^T^, LB39^T^ and LB41, respectively (Table S2; Fig. 3); the hybrid system NRPS-PKS was only found in isolates LB39^T^ and LB41. Secondary metabolite related genes detected using the SEED server were also discontinuously distributed, as exemplified by genes associated with lanthionine synthases which varied from 4 in isolate LB4 to 16 in isolate LB39^T^ (Table 5) while all of the isolates, apart from strain LB32^T^, contained genes related to the synthesis of thiazole-oxazole-modified microcins, ribosomally produced peptides with post-translationally installed heterocycles derived from cysteine, serine and threonine residues^68^. In turn, only the genome of isolate LB19 harboured a gene associated with the synthesis of clavulanic acid, which encodes for a clavaldehyde dehydrogenase (contig 9) according to a RAST analysis. In this context it is also interesting that compounds extracted from the isolates varied in their ability to inhibit a variety of indicator micro-organisms and the HepG2 cells (Table 4).

**Table 5:** Genes implicated in secondary metabolism in the strains of study and closest type strains detected by RAST subsystems.

		Isolate LB4	Isolate LB19	Isolate LB32^T^	Isoalte LB39^T^	Isolate LB41	*M*. *chalcea* DSM 43026^T^	*M*. *saelicesensis* Lupac 09^T^
**Secondary metabolism**		9	10	6	19	17	10	4
Thiazole- oxazole-modified microcin (TOMM) synthesis		5	4	0	3	7	3	0
TOMM biosynthesis dehydrogenase (protein B)		1	1	0	1	2	1	0
TOMM biosynthesis cyclodehydratase (protein C)		2	1	0	1	2	1	0
TOMM biosynthesis docking scaffold (protein D)		2	1	0	1	2	1	0
FIG214983: hypothetical protein		0	0	0	0	1	0	0
SagD family docking scaffold		0	1	0	0	0	0	0
Lanthionine synthetases		4	5	6	16	6	7	4
LanB	Lanthionine biosynthesis protein LanB	1	1	0	2	1	1	0
LanC	Lanthionine biosynthesis cyclase LanC	0	1	0	2	0	1	0
MT	*O*-methyltransferase clustered with LanBC	1	0	0	1	1	0	0
IsoAspMT	Protein-L-isoaspartate *O*-methyltransferase (EC 2.1.1.77)	2	2	3	8	3	3	1
LanL	Lanthionine biosynthesis protein LanL	0	1	0	1	1	1	1
LanM	Lanthionine biosynthesis protein LanM	0	0	3	0	0	0	2
HP1	Hypothetical protein associated with LanBC	0	0	0	2	0	1	0
Clavulanic acid biosynthesis		0	1	0	0	0	0	0
CAD	Clavaldehyde dehydrogenase	0	1	0	0	0	0	0

The genomes of the *M*. *chalcea* strains were found to harbour 38 different BGCs that presented some similarity with known compounds, out of which 10 have not been detected previously in *Micromonospora* strains; 7 of these BGCs were only present in the genome of isolate LB41 and the other three in the other LB strains (LB4, LB32^T^, LB39^T^ and LB41). In addition, 26 out of the 38 BGCs were only found in the genomes of one out of the three *M*. *chalcea* strains, 9 in two of them and the remaining three in all of them. These results provide further evidence that *M*. *chalcea* strains are a good source of novel antibiotics, notably aminoglycosides, lactones and macrolides^61^. However, none of the *M*. *chalcea* strains had the capacity to synthesize tetrocarcin A, a spirotetronate antibiotic produced by *M*. *chalcea* NRRL 11289^69^ or chalcidin or neomycin produced by *M*. *chalcea* sp.^70^ and *M*. *chalcea* B9-683^71^, respectively though the taxonomic provenance of these strains is questionable.

The results of this study taken together with those reported by Carro *et al*.^16^ show that the genomes of *Micromonospora* strains are a unique source of BGCs that have the potential to synthesise an array of completely novel and uncharacterised specialised metabolites. It is particularly interesting that the genomes of the novel micromonosporae from the extreme hyper-arid Lomas Bayas soil have the capacity to synthesise a broad range of new bioactive compounds. It is also encouraging that *M*. *chalcea* strains LB4 and LB41 showed moderate to pronounced antitumour activity and that *M*. *ureilytica* strain LB19 and the putative type strains of *M*. *arida* (LB32^T^) and *M*. *inaquosa* (LB39^T^) showed promise in restricting the growth of the MRD *K*. *pneumonia*e strain. Gifted actinobacterial isolates such as these have a role to play in the search and discovery of new chemical scaffolds using state-of-the-art genome tools^9^, including ones designed to induce the expression of silent BGCs^31,72^. Indeed, novel micromonosporae should feature much more prominently in the search and discovery of new classes of specialised metabolites that are needed to control MRD pathogens which currently threaten to take humankind back to the pre-antibiotic days of medicine^73,74^. The search for additional novel and rare gifted micromonosporae from Atacama Desert habitats should include functional metagenomics and the use of isolation procedures known to target members of this taxon^26,60,61^ and improved characterisation procedures, notably ones for acquiring reliable phenotypic data^56^.

### Stress-related genes encoded in the genome of the strains

The genomes of all of the isolates contained between 97 and 131 putative genes known to be associated with stress responses, notably ones coding for carbon starvation, heat shock responses, osmoregulation and oxidative stress (Table S3). The genomes of isolates LB19, LB32^T^ and LB39^T^ contained *osm*Y the expression of which is known to be induced under hyperosmotic stress^75^. The expression of this gene is associated with the induction of the glycine betaine binding protein (*pro*U), which was found in all of the isolated strains (with an average of 4 genes). The genomes of isolates LB19, LB32^T^ and LB41 harboured genes involved in mycothiol biosynthesis (*msh*A, *msh*B, *msh*C, *msh*D), an analogue of glutathione that acts as an electron acceptor/donor and serves as a cofactor in detoxification reactions for alkylating agents, free radicals and xenobiotics^76^. Catalase and peroxidase genes were found in all the genomes confirming the results of the laboratory tests (Table S3). However, the type strain of *M*. *chalcea*, which gave a negative result has the capacity to produce catalase^16^. The genomes of all the isolates encode for several RNA polymerase Sigma factors and serine phosphatases that acts as regulators, it is known that Sigma B controls a general stress regulon which is induced when cells encounter growth-limiting conditions^77^.

The world’s highest levels of surface ultraviolet (UV) irradiance have been reported from the Atacama Desert^78^ hence it is particularly interesting that the genomes of all of the isolates included genes associated with protection against UV-radiation; we have previously shown that these strains grew on M65 agar following exposure to UV light at 100 mJoules/second for 30 minutes^26^. The genomes of all of the isolates contained genes belonging to the uvrABC DNA repair system, associated with excision proteins which have been reported in several bacteria^79^. Specific desiccation stress genes were not detected in any of the genomes though several genes associated with the biosynthesis and uptake of trehalose were present, this sugar has been linked with tolerance to heat and desiccation in bacteria^80^. The assortment of stress related genes outlined above provide an insight into how micromonosporae are able to adapt to severe environmental conditions that prevail in arid Atacama Desert soils. However, a similar complement of stress related genes have been found in the genomes of representative *Micromonospora* taxa isolated from diverse habitats^16^ thereby supporting the view that micromonoporae *per se* have the capacity to colonize multiple microhabitats^28^, including ones associated with extreme biomes^58,81^. In this context it is also interesting that the genomes of all of the isolates contained genes associated with the production of a range of growth promoters of potential value in phytostimulation^16^. The genomes of all of the isolates also contained methylglyoxal detoxification genes (*glo*A and *glo*B) which are associated with increases in plant tolerance to abiotic and biotic stress^82^.

### Description of ***Micromonospora arida*** sp. nov

*Micromonospora arida* (a’ri.da L. fem. adj. *arida*, dry, referring to the isolation of the strain from an extreme hyper-arid soil).

Aerobic, Gram-stain-positive, chemoorganotrophic actinobacterium which produces non-motile, single spores on extensively branched substrate hyphae, but does not form aerial hyphae. Colonies are orange on ISP2 agar turning brown-black on sporulation. Grows between 20–37 °C, optimally ~28 °C, at pH 7.0 and 8.0, optimally ~pH 7.0, and in the presence of 1% w/v sodium chloride. Casein and starch are degraded. Catalase positive and oxidase negative. Cystine arylamidase, esterase (C4), esterase lipase (C8), N-acetyl-β-glucosaminidase, α- and β-galactosidase, α- and β-glucosidase, β-glucuronidase, leucine arylamidase, lipase (C14), naphthol-AS-BI-phosphohydrolase, trypsin and valine arylamidase are produced, but not acid or alkaline phosphatase, α-chymotrypsin, α-fucosidase or α-mannosidase. Metabolises L-alanine, L-arginine and L-serine (amino-acids); acetic acid, acetoacetic acid, L-aspartic acid, β-*hydroxy*-butyric acid, D-galacturonic acid, D-gluconic acid, L-glutamic acid, D-glucoronic acid, α-*keto*-glutaric acid, D-lactic acid methyl ester, L-malic acid, N-acetyl-neuraminic acid, methyl pyruvate, propionic acid, L-pyroglutamic acid, D- saccharic acid and bromo-succinic acid (organic acids); D-cellobiose, D-fructose, D-fructose-6-phosphate, L-fucose, D-galactose, D-glucose, D-glucose-6-phosphate, N-acetyl-D-galactosamine, N-acetyl-D-glucosamine, 3-O-methyl-D-glucose, glycerol, *myo*-inositol, D-mannitol, N-*acetyl*-B-D-mannose, D-salicin, D-sorbitol, stachyose, sucrose, D-trehalose and D-turanose (sugars) and dextrin, gelatin and pectin (polymers), but not D-aspartic acid, L-histidine or D-serine (amino acids), γ-amino-*n*-butyric acid, butyric acid, α- and α-*keto*-butyric acid, citric acid, D-malic acid, L-galacturonic acid-γ-lactate, L-lactic acid, mucic acid, p-*hydroxy-*phenylacetic acid and quinic acid (organic acids), or D-arabitol, D-fucose, glucoranimide or α-D-lactose (sugars). Resistant to aztreonam, nalidixic acid and rifamycin SV, but sensitive to fusidic acid, minocycline, troleandomycin and vancomycin. Does not grow at pH 5.0 or in the presence of guanidine hydrochloride, inosine, lithium chloride, potassium tellurite, sodium bromate, sodium lactate, tetrazolium blue or tetrazolium violet. Whole cell hydrolysates contain *hydroxy*- and *meso*-A2pm, galactose, glucose, mannose, rhamnose, ribose and xylose. The major fatty acids are *iso*-C_15:0_, *iso*-C_17:0_, *anteiso*-C_17:0_ and C_17:0_ and the predominant isoprenologues MK-10 (H_4_, H_6_, H_8_); the polar lipid profile contains diphosphatidylglycerol, phosphatidylethanolamine and phospatidylinositol together with unidentified components. The dDNA G + C content is 71.0 mol% and the genome size ~7.1 Mbp.

The type strain, LB32^T^ (=CECT 9662^T^ = LMG 30765^T^) was isolated from an extreme hyper-arid surface soil (2 cm) collected from the Lomas Bayas region of the Atacama Desert soil in Chile. The genome accession number is QGSY00000000.

### Description of *Micromonospora inaquosa* sp. nov

*Micromonospora inaquosa* (in.a.quo’sa. L. fem. adj. *inaquosa* without water, referring to the isolation of the strain from an extreme hyper-arid soil).

Aerobic, Gram-stain-positive, chemoorganotrophic actinobacterium which produces non-motile, single spores on extensively branched substrate hyphae, but does not form aerial hyphae. Colonies are orange on ISP2 agar turning brown-black on sporulation. Grows between 12–37 °C, optimally ~28 °C, at pH 7.0 and 8.0, optimally ~pH 7.0 and in the presence of 1% w/v sodium chloride. Casein and starch are degraded. Catalase and oxidase positive. Acid and alkaline phosphatase, α-chymotrypsin, cystine arylamidase, esterase (C4), esterase lipase (C8), N-acetyl-β-glucosaminidase, α- and β-galactosidase, α- and β-glucosidase, β-glucuronidase, leucine arylamidase, lipase (C14), α-mannosidase, naphthol-AS-BI-phosphohydrolase, trypsin, and valine arylamidase are produced, but not α-fucosidase. Metabolises L-alanine, L-arginine, and D- and L-serine (amino-acids); acetic acid, acetoacetic acid, L-aspartic acid, butyric acid, D-galacturonic acid, D-gluconic acid, L-glutamic acid, D-glucuronic acid, D-lactic acid methyl ester, L-malic acid, methyl pyruvate, propionic acid, D- saccharic acid and bromo-succinic acid (organic acids); D-cellobiose, D-fructose, D-fructose-6-phosphate, L-fucose, D-galactose, D-glucose, D-glucose-6-phosphate, N-acetyl-D-galactosamine, N-acetyl-D-glucosamine, 3-O-methyl-D-glucose, glycerol, N-acetyl-β-D-mannose, D-salicin, stachyose, sucrose, D-trehalose and D-turanose (sugars) and dextrin and gelatin (polymers), but not L-histidine (amino acids); D-aspartic acid, γ-amino-*n*-butyric acid, α- and β-*hydroxy*-butyric acid, α-*keto*-butyric acid, citric acid, α-*keto*-glutaric acid, D-malic acid, L-galacturonic acid-γ-lactate, L-lactic acid, mucic acid, p-*hydroxy-*phenyl acetic acid, L-pyroglutamic acid, and quinic acid (organic acids); pectin (polymer) or glucoranimide, *myo*-inositol, D-mannitol or D-sorbitol (sugars). Resistant to aztreonam, minocycline, nalidixic acid and rifamycin SV, but sensitive to fusidic acid, troleandomycin and vancomycin. Does not grow at pH 5.0 or in the presence of guanidine hydrochloride, inosine, lithium chloride, potassium tellurite, sodium bromate, sodium lactate, tetrazolium blue or tetrazolium violet. Whole cell hydrolysates contain *hydroxy*- and *meso*-A2pm, galactose, glucose, mannose, rhamnose, ribose and xylose. The major fatty acids are *iso*-C_15:0_, *iso*-C_16:0_, 10-methyl C_17:0_ and C_17:0_, the predominant isoprenologues MK-10 (H_4_, H_6_, H_8_); the polar lipid profile consists of diphosphatidylglycerol, phosphatidylethanolamine together with unidentified components. The dDNA G + C content is 70.6 mol% and the genome size ~7.8 Mbp.

The type strain, LB39^T^ (=CECT 9663^T^ = LMG 30766^T^) was isolated from an extreme hyper-arid surface soil (2 cm) collected from the Lomas Bayas region of the Atacama Desert soil in Chile. The genome accession number is QGSZ00000000.

## Methods

### Selective isolation

All of the strains (isolates LB4, LB19, LB32^T^, LB39^T^ and LB41) were recovered from the surface (2 cm) of an extreme hyper-arid soil collected from the Lomas Bayas region of the Atacama Desert (23° 24′ 27″ S, 69°31′03″ W 24.02.2014) by Professor Luis Cáceres (University of Antofagasta) as previously described^26^. Briefly, when transferred to the UK the sample was stored at 4 °C. The strains were isolated using the selective isolation procedure devised by Makkar and Cross^83^; to this end, aliquots (100 µl) of the 10^−1/2^ and 10^−1^ dilutions of the soil in ¼ strength Ringer’s solution were spread over the surface of starch-casein agar plates^84^ supplemented with sterile cycloheximide, nystatin and novobiocin (each at 25 µg/ml). Three replicate plates were prepared per dilution and incubated at 28 °C for 3 weeks when five characteristic orange-coloured *Micromonospora* colonies were detected. The isolates were maintained on M65 (DSMZ medium) agar plates and as mixtures of hyphal fragments and spores in 20% v/v glycerol at −80 °C.

### Extraction of DNA and determination of RAPD profiles

Genetic profiles were generated by PCR using the primer M13 (5′-GAGGGTGGCGGTTCT-3′)^85^. DNA was extracted from all of the isolates using a REDExtract-N.Amp kit (Sigma) and amplified following the manufacturer’s recommendations to give a final volume of 20 μl per reaction; the thermal cycling parameters were: 7 min at 95 °C, 35 cycles of 1 min at 94 °C, 1 min at 45 °C and 2 min at 72 °C, followed by a 6 min final extension at 72 °C. A 1.5% agarose gel containing ethidium bromide was loaded with 5 µl of each of the PCR products and electrophoresis run at 85 V for 90 minutes in freshly prepared 1x TBE-EDTA buffer at pH 8.0 using a Bio-Rad PowerPac 300 power supply; a DNA molecular weight marker (1 kbp) was used as a molecular size standard. Photographs of the electrophoresis results recovered as TIFF files were aligned using BioNumerics package 6.0 into similarity groups.

### Phylogenetic analysis

Genomic DNA extraction, PCR-mediated amplification and 16S rRNA gene sequencing were performed as described by Carro *et al*.^26^. Universal primers 27 F and 1522R^86^ were used for PCR amplification in a final volume of 50 μl using Bioline 2x MiFi^TM^ mix following instructions of the manufacturer. The PCR products were purified and sequenced using the EZseq Barcode Service (Macrogen). The manually aligned sequences were compared with those of their closest neighbours retrieved from the EzBioCloud server^87^. Maximum-likelihood^88^ and neighbour-joining^89^ algorithms were used to generate the phylogenetic trees. In addition, a multilocus sequence analysis (MLSA) based on 16S rRNA, *atp*D, *gyr*B, *rec*A and *rpo*B gene sequences retrieved from whole-genome sequences of the isolates was carried out using established procedures^52^ and a micromonosporal MLSA tree generated from the 9165 nucleotides using the neighbour-joining and maximum-likelihood algorithms.

### Phenotypic profiles

The isolates were examined for micromorphological, Gram-stain and motility using a phase-contrast microscope (Leica; CTR MIC) and 7-day-old cultures grown on GYM *Streptomyces* agar (DSMZ medium 65^90^). They were also examined for their ability to grow in the presence of various concentrations of sodium chloride (1, 2, 5, 7 and 9% w/v) and over a range of pH (4.0–9.0 at one unit intervals) and temperature regimes (4, 10, 20, 28 37 and 40 °C) using GYM as the basal medium. All of these tests were recorded on duplicated cultures after 14 days of incubation. Enzymatic activities of the isolates were determined using API ZYM kits (bioMerieux) according to the manufacturer’s instructions. The ability of the isolates to oxidise diverse carbon and nitrogen sources and to show resistance to inhibitory compounds was determined using GEN III microplates in an Omnilog device (BIOLOG Inc., Haywood, USA) and the exported data of the duplicated samples analysed using the opm package for R version 1.06^91,92^. Other phenotypic analyses were determined following Carro *et al*.^93^.

Biomass for the chemotaxonomic analyses carried out on each of the isolates was prepared in shake flasks (180 rpm) in ISP2 broth^94^ following incubation at 28 °C for 14 days, washed twice in sterile saline solution, and freeze-dried. Standard procedures were used to detect the isomers of diaminopimelic acid (A_2_pm)^95^, menaquinones^96^, polar lipids^97^ and whole cell sugar composition^98^, using appropriate controls. Cellular fatty acids were extracted, methylated, examined by gas chromatography (Agilent Technologies mod. 7890 A) and analysed using the protocol of the Sherlock Microbial Identification (MIDI) system, version 6.3^99^. The resultant peaks were named using the RTSBA6 database.

### Whole-genome sequencing and genomic analyses

A single colony of each of the isolates was used to inoculate 50 ml aliquots of M65 broth and the resultant preparations incubated at 28 °C for 7 days when cells were centrifuged prior to sending to Microbes NG (Birmingham, UK). Genomic DNA extracted from each of the preparations was sequenced on an Illumina HiSeq 2500 instrument with 2 × 250 bp paired-end reads. All of the strains were analysed using a standard pipeline and identified with their closest reference genome using Kraken^100^ and by mapping the reads using BWA-MEM^101^. The reads were assembled into contigs using SPAdes 3.90^102^ and contigs <500 bp discarded. Variant calling performed on the draft assemblies using VarScan were reordered and reoriented relative to a reference genome based on a MUMmer whole-genome alignment. An automated annotation was performed using Prokka^103^ while antiSMASH 4.0 was used to determine and compare BGCs encoding for natural products^104^. The presence of other genes was detected using the SEED viewer^105^ following RAST annotation of the genomes^106,107^.

Digital DNA:DNA hybridisation (dDDH) values between the genomes of the isolates and between them and available genomes of their phylogenetic neighbours were calculated using the genome-to-genome distance calculator, GGDC 2.0, using formula 2 of the GGDC web server available at http://ggds.dsmz.de/ggdc.php. In addition, ANI values were determined between the strains using OAT version 0.93.1^108^.

### Bioassays with the extract of the isolates

Each of the isolates was shaken in 50 ml of ISP 2 broth^94^ at 180 revolutions per minute (rpm) with resin beads (Amberlite XAD-16N, Sigma) at 28 °C for 14 days. Each preparation was centrifuged (4100 rpm) for 15 min and the biomass and XAD-16N resin beads soaked overnight in methanol and filtered through glass wool prior to evaporation of the methanol fraction at 40 °C by nitrogen sparging to generate the extracts. They were fractionated with Solid Phase Extraction (SPE) cartridges, using either 2 g or 5 g of a C18 resin (55 µm, 70 Å, from Strata) depending on the weight of the extract; four column volumes of the following solvents were sequentially used for the fractionation of the samples: 100% water, 25%, 50, 75 and 100% methanol and 100% methanol + 0.01% TFA. The eluted fractions were screened using liquid chromatography–mass spectrometry (LCMS).

Inhibition tests were carried out on each of the fractions using a concentration of 300 µg/ml in 96 well-plates containing a total incubation volume of 200 µl. The screening assays were carried out using a range of indicator microorganisms, namely MRD strains of *Acinetobacter baumannii* (CL5973), *Escherichia coli* (ATCC 25922), *Klebsiella pneumoniae* (ATCC 700603), *Pseudomonas aeruginosa* (MB5919), *Staphylococcus aureus* MB5393 (methicillin-resistant) and ATCC 29213 (methicillin-sensitive), as well as MDR *Aspergillus fumigatus* ATCC 46645. Negative controls were included in the plates for each microorganism tested without extracts. Anti-tumour activity against human hepatocellular carcinoma HepG2 cells was determined in the same system using concentrations of 75 mg/ml for each fraction. Negative and positive controls were included containing dimethyl sulfoxide (DMSO) and methyl methane sulphonate (MMS), respectively. Dexorubicin was used as standard at several concentrations (0.11, 0.34, 1, 3, 9, 28, 83, 250 μg/l).

Accession numbers for NCBI deposited genome sequences: LB4 (QGSX00000000), LB19 (QDGB00000000), LB32^T^ (QGSY00000000), LB39^T^ (QGSZ00000000), LB 41 (QGTA00000000) and SRA accession numbers are: LB4 (SRR8278219), LB19 (SRR8278244), LB32^T^ (SRR8278835), LB39^T^ (SRR8278845), LB41 (SRR8278854).

## Supplementary information

Supplementary data
