# Supplementary material for: Uncovering the potential of novel micromonosporae isolated from an extreme hyper-arid Atacama Desert soil
Source: Sci Rep. 2019 Mar 18;9:4678. doi: 10.1038/s41598-019-38789-z (PMC6423291; doi:10.1038/s41598-019-38789-z)

Uncovering the potential of novel micromonosporae isolated from an extreme hyper-arid Atacama Desert soil

Lorena Carro[1,2*], Jean Franco Castro[1,3], Valeria Razmilic[1,3], Imen Nouioui[1], Che Pan[1], José M. Igual[4], Marcel Jaspars[5], Michael Goodfellow[1], Alan T. Bull[6], Juan A. Asenjo[3], Hans-Peter Klenk[1]

[1] School of Natural and Environmental Sciences, Newcastle University, Newcastle-upon Tyne, UK.

[2] Microbiology and Genetics Department, University of Salamanca. Salamanca, Spain.

[3] Centre for Biotechnology and Bioengineering (CeBiB), Department of Chemical Engineering, Biotechnology and Materials, Universidad de Chile, Beauchef 851, Santiago, Chile.

[4] Instituto de Recursos Naturales y Agrobiología de Salamanca, Consejo Superior de Investigaciones Científicas (IRNASA-CSIC), Salamanca, Spain.

[5] Marine Biodiscovery Centre, Department of Chemistry, University of Aberdeen, Scotland, UK

[6] School of Biosciences, University of Kent, Canterbury, UK

* lcg@usal.es

Table S1. MLSA distances showing relationships between the isolates and between them and closely related *Micromonospora* type strains.

	M. aurantiaca ATCC 27029^T	*M. auratinigra* DSM 44815^T	*M. chaiyaphumensis* DSM 45246^T	*M. chalcea* DSM 43026^T	*M. chersina* DSM 44151^T	*M. chokoriensis* DSM 45160^T	*M. citrea* DSM 43903^T	*M. coriariae* DSM 44875^T	*M. cremea* DSM 45599^T	*M. echinaurantiaca* DSM 43904^T	*M. echinofusca* DSM 43913^T	*M. endolithica* AA-459^T	*M. humi* DSM 45647^T	*M. krabiensis* DSM 45344^T	*M. lupini* Lupac 08	*M. marina* DSM 45555^T	*M. nigra* DSM 43818^T	*M. noduli* GUI43^T	*M. peucetia* DSM 43363^T	*M. saelicesensis* Lupac 09^T	*M. sediminicola* DSM 45794^T	*M. tulbaghiae* DSM 45142^T	*M. ureilityca* GUI23^T	*M. zamorensis* DSM 45600^T	**Isolate LB4**	**Isolate LB19**	**Isolate LB32^T**	**Isolate LB39^T**	**Isolate LB41**
M. aurantiaca ATCC 27029^T																													
M. auratinigra DSM 44815^T	0,036																												
M. chaiyaphumensis DSM 45246^T	0,036	0,032																											
M. chalcea DSM 43026^T	0,014	0,037	0,037																										
M. chersina DSM 44151^T	0,037	0,032	0,018	0,037																									
M. chokoriensis DSM 45160^T	0,056	0,053	0,056	0,060	0,054																								
M. citrea DSM 43903^T	0,044	0,042	0,039	0,047	0,039	0,052																							
M. coriariae DSM 44875^T	0,056	0,053	0,052	0,057	0,050	0,042	0,051																						
M. cremea DSM 45599^T	0,058	0,053	0,054	0,060	0,050	0,036	0,049	0,021																					
M. echinaurantiaca DSM 43904^T	0,044	0,043	0,038	0,046	0,039	0,049	0,033	0,049	0,047																				
M. echinofusca DSM 43913^T	0,046	0,045	0,041	0,049	0,043	0,052	0,028	0,051	0,050	0,035																			
M. endolithica AA-459^T	0,048	0,044	0,044	0,049	0,042	0,053	0,043	0,050	0,050	0,045	0,044																		
M. humi DSM 45647^T	0,035	0,039	0,041	0,036	0,042	0,060	0,051	0,061	0,061	0,050	0,055	0,052																	
M. krabiensis DSM 45344^T	0,050	0,051	0,044	0,053	0,044	0,051	0,044	0,055	0,053	0,036	0,049	0,055	0,056																
M. lupini Lupac 08	0,056	0,057	0,058	0,060	0,058	0,029	0,058	0,039	0,038	0,052	0,054	0,057	0,061	0,055															
M. marina DSM 45555^T	0,016	0,037	0,037	0,019	0,036	0,060	0,045	0,059	0,060	0,045	0,050	0,049	0,036	0,055	0,061														
M. nigra DSM 43818^T	0,055	0,051	0,051	0,060	0,051	0,062	0,050	0,060	0,061	0,051	0,050	0,051	0,059	0,060	0,066	0,058													
M. noduli GUI43^T	0,060	0,057	0,061	0,063	0,061	0,031	0,059	0,036	0,039	0,055	0,055	0,057	0,065	0,057	0,023	0,064	0,066												
M. peucetia DSM 43363^T	0,050	0,047	0,046	0,053	0,046	0,052	0,033	0,049	0,047	0,039	0,025	0,047	0,056	0,050	0,055	0,054	0,055	0,055											
M. saelicesensis Lupac 09^T	0,059	0,057	0,060	0,062	0,059	0,029	0,058	0,034	0,037	0,055	0,055	0,057	0,065	0,057	0,021	0,063	0,065	**0,007**	0,054										
M. sediminicola DSM 45794^T	0,023	0,032	0,033	0,023	0,034	0,058	0,043	0,057	0,060	0,044	0,047	0,046	0,030	0,050	0,059	0,024	0,054	0,063	0,051	0,063									
M. tulbaghiae DSM 45142^T	0,016	0,036	0,035	0,019	0,035	0,061	0,045	0,057	0,059	0,046	0,048	0,048	0,036	0,054	0,060	0,019	0,056	0,063	0,051	0,063	0,026								
M. ureilityca GUI23^T	0,054	0,051	0,052	0,057	0,051	0,028	0,056	0,041	0,039	0,052	0,050	0,055	0,058	0,052	0,023	0,058	0,061	0,017	0,051	0,017	0,057	0,058							
M. zamorensis DSM 45600^T	0,061	0,060	0,063	0,064	0,063	0,031	0,060	0,040	0,039	0,058	0,059	0,060	0,066	0,059	0,028	0,066	0,068	0,025	0,058	0,023	0,064	0,065	0,021						
**Isolate LB4**	0,013	0,037	0,037	**0,002**	0,037	0,060	0,047	0,057	0,060	0,046	0,049	0,049	0,036	0,053	0,060	0,019	0,059	0,063	0,053	0,062	0,023	0,018	0,057	0,064					
**Isolate LB19**	0,060	0,056	0,060	0,062	0,059	0,029	0,058	0,035	0,034	0,055	0,056	0,055	0,065	0,057	0,024	0,063	0,065	0,018	0,054	0,016	0,061	0,063	**0,005**	0,022	0,062				
**Isolate LB32^T**	0,058	0,056	0,059	0,061	0,058	0,027	0,057	0,036	0,038	0,054	0,055	0,055	0,064	0,056	0,022	0,062	0,064	0,011	0,055	0,008	0,062	0,061	0,016	0,023	0,061	0,016			
**Isolate LB39^T**	0,056	0,056	0,057	0,059	0,058	0,032	0,055	0,036	0,041	0,051	0,054	0,054	0,061	0,054	0,028	0,060	0,063	0,025	0,055	0,024	0,058	0,059	0,030	0,031	0,059	0,026	0,024		
**Isolate LB41**	0,013	0,037	0,037	**0,002**	0,037	0,060	0,047	0,057	0,060	0,046	0,049	0,049	0,036	0,053	0,060	0,019	0,059	0,063	0,053	0,062	0,023	0,018	0,057	0,064	**0,000**	0,062	0,061	0,059	

Table S2. Bioclusters detected in the genomes of the *Micromonospora* isolates using antiSMASH.

***Micromonospora* sp. LB4**

Cluster	Type	Most similar known cluster	MIBiG BGC-ID
Cluster 1	Lantipeptide	-	-
Cluster 2	Siderophore	Desferrioxamine_B_biosynthetic_gene_cluster (80% of genes show similarity)	BGC0000941_c1
Cluster 3	Cf_putative	-	-
Cluster 4	Cf_fatty_acid	-	-
Cluster 5	Cf_fatty_acid	-	-
Cluster 6	Terpene	-	-
Cluster 7	Nrps	-	-
Cluster 8	Other	Diazepinomicin_biosynthetic_gene_cluster (12% of genes show similarity)	BGC0000679_c1
Cluster 9	Cf_putative	Coumermycin_A1_biosynthetic_gene_cluster (12% of genes show similarity)	BGC0000833_c1
Cluster 10	Cf_putative	-	-
Cluster 11	Nrps	-	-
Cluster 12	Cf_putative	-	-
Cluster 13	Terpene	Lymphostin_biosynthetic_gene_cluster (25% of genes show similarity)	BGC0001006_c1
Cluster 14	Cf_saccharide	-	-
Cluster 15	Cf_putative	Svaricin_biosynthetic_gene_cluster (6% of genes show similarity)	BGC0001382_c1
Cluster 16	Cf_putative	-	-
Cluster 17	Cf_putative	Diazepinomicin_biosynthetic_gene_cluster (20% of genes show similarity)	BGC0000679_c1
Cluster 18	Terpene	-	-
Cluster 19	Nrps	-	-
Cluster 20	T3pks	-	-
Cluster 21	Cf_fatty_acid	-	-
Cluster 22	Otherks	Naphthyridinomycin_biosynthetic_gene_cluster (7% of genes show similarity)	BGC0000394_c1
Cluster 23	Other	-	-
Cluster 24	Cf_putative	-	-
Cluster 25	Other	Microcystin_biosynthetic_gene_cluster (30% of genes show similarity)	BGC0001016_c1
Cluster 26	Cf_putative	-	-
Cluster 27	Cf_putative	Phosphonoglycans_biosynthetic_gene_cluster (5% of genes show similarity)	BGC0000806_c1
Cluster 28	T2pks	Frankiamicin_biosynthetic_gene_cluster (21% of genes show similarity)	

***Micromonospora* sp. LB19**

Cluster	Type	Most similar known cluster	
Cluster 1	T2pks	Pradimicin_biosynthetic_gene_cluster (21% of genes show similarity)	BGC0001394_c1
Cluster 2	Cf_putative	-	BGC0000026_c1
Cluster 3	Lassopeptide	-	BGC0000202_c1
Cluster 4	Cf_putative	Lobosamide_biosynthetic_gene_cluster (6% of genes show similarity)	BGC0000679_c1
Cluster 5	Cf_putative	-	-
Cluster 6	Head_to_tail	-	BGC0000394_c1
Cluster 7	Cf_putative	-	BGC0000679_c1
Cluster 8	Cf_putative	Fengycin_biosynthetic_gene_cluster (20% of genes show similarity)	BGC0000354_c1
Cluster 9	Cf_putative	-	-
Cluster 10	Cf_saccharide	-	BGC0000807_c1
Cluster 11	Cf_putative	Clarexpoxcin_biosynthetic_gene_cluster (10% of genes show similarity)	-
Cluster 12	Cf_putative	-	-
Cluster 13	Cf_saccharide	Coumermycin_A1_biosynthetic_gene_cluster (12% of genes show similarity)	-
Cluster 14	Siderophore	-	BGC0000078_c1
Cluster 15	Cf_fatty_acid	Alnumycin_biosynthetic_gene_cluster (6% of genes show similarity)	
Cluster 16	Cf_putative	Friulimicin_biosynthetic_gene_cluster (6% of genes show similarity)	-
Cluster 17	Cf_putative	-	-
Cluster 18	Cf_putative	-	-
Cluster 19	Cf_putative	-	BGC0001011_c1
Cluster 20	Terpene	Phosphonoglycans_biosynthetic_gene_cluster (3% of genes show similarity)	BGC0000279_c1
Cluster 21	Cf_putative	Pyrrolomycin_biosynthetic_gene_cluster (5% of genes show similarity)	BGC0001036_c1
Cluster 22	Cf_saccharide	-	-
Cluster 23	Cf_putative	-	-
Cluster 24	Cf_putative	Meilingmycin_biosynthetic_gene_cluster (2% of genes show similarity)	-
Cluster 25	T3pks	Alkyl-O-Dihydrogeranyl-Methoxyhydroquinones_biosynthetic_gene (71% of genes show similarity)	BGC0000963_c1
Cluster 26	Cf_fatty_acid	Paromomycin_biosynthetic_gene_cluster (5% of genes show similarity)	**MIBiG BGC-ID**
Cluster 27	Cf_fatty_acid	Avilamycin_A_biosynthetic_gene_cluster (5% of genes show similarity)	BGC0001007_c1
Cluster 28	Cf_putative	-	BGC0000705_c1
Cluster 29	Cf_putative	Kanamycin_biosynthetic_gene_cluster (15% of genes show similarity)	-
Cluster 30	Cf_putative	-	BGC0000087_c1
Cluster 31	Lantipeptide	-	BGC0001303_c1
Cluster 32	Thiopeptide	K-252a_biosynthetic_gene_cluster (5% of genes show similarity)	BGC0000609_c1
Cluster 33	Nrps	Albachelin_biosynthetic_gene_cluster (90% of genes show similarity)	BGC0000100_c1
Cluster 34	Other	Herboxidiene_biosynthetic_gene_cluster (2% of genes show similarity)	BGC0000141_c1
Cluster 35	Cf_putative	Actagardine_biosynthetic_gene_cluster (6% of genes show similarity)	BGC0000706_c1
Cluster 36	Lantipeptide	Catenulipeptin_biosynthetic_gene_cluster (60% of genes show similarity)	-
Cluster 37	Terpene	-	BGC0001077_c1
Cluster 38	Cf_putative	-	-
Cluster 39	Cf_putative	Kanamycin_biosynthetic_gene_cluster (15% of genes show similarity)	-
Cluster 40	Cf_putative	Esmeraldin_biosynthetic_gene_cluster (8% of genes show similarity)	BGC0000132_c1
Cluster 41	Cf_putative	-	-
Cluster 42	Phosphonate	Gentamicin_biosynthetic_gene_cluster (4% of genes show similarity)	BGC0000105_c1
Cluster 43	Cf_putative	-	-
Cluster 44	Cf_putative	-	-
Cluster 45	Terpene	Sioxanthin_biosynthetic_gene_cluster (100% of genes show similarity)	-
Cluster 46	Cf_putative	-	BGC0000833_c1
Cluster 47	Cf_putative	Teichomycin_biosynthetic_gene_cluster (11% of genes show similarity)	BGC0000340_c1
Cluster 48	Bacteriocin	Lymphostin_biosynthetic_gene_cluster (33% of genes show similarity)	
Cluster 49	Cf_putative	-	-
Cluster 50	Cf_putative	-	-
Cluster 51	Cf_putative	Asukamycin_biosynthetic_gene_cluster (4% of genes show similarity)	BGC0000137_c1
Cluster 52	Cf_putative	-	-

Cluster 53	Terpene	-	BGC0000806_c1
Cluster 54	Cf_putative	Sanglifehrin_A_biosynthetic_gene_cluster (4% of genes show similarity)	-
Cluster 55	Cf_putative	-	-
Cluster 56	Cf_putative	-	-
Cluster 57	Cf_fatty_acid-T1pks	Pyrrolomycin_biosynthetic_gene_cluster (34% of genes show similarity)	BGC0001172_c1
Cluster 58	Cf_putative	-	BGC0000148_c1
Cluster 59	Cf_putative	-	
Cluster 60	Cf_putative	Kinamycin_biosynthetic_gene_cluster (8% of genes show similarity)	-
Cluster 61	Cf_putative	-	-
Cluster 62	Siderophore	-	BGC0000033_c1
Cluster 63	Cf_putative	-	BGC0000806_c1
Cluster 64	Cf_saccharide	Polysaccharide_B_biosynthetic_gene_cluster (10% of genes show similarity)	-
			BGC0001087_c4
			-

***Micromonospora* sp. LB32**

Cluster	Type	Most similar known cluster	MIBiG BGC-ID
Cluster 1	Cf_putative	-	-
Cluster 2	Cf_putative	-	-
Cluster 3	Cf_putative	-	-
Cluster 4	Cf_putative	WS9326_biosynthetic_gene_cluster (5% of genes show similarity)	BGC0001297_c1
Cluster 5	Bacteriocin	Lymphostin_biosynthetic_gene_cluster (33% of genes show similarity)	BGC0001006_c1
Cluster 6	Cf_putative	-	-
Cluster 7	Cf_putative	-	-
Cluster 8	Cf_putative	-	-
Cluster 9	Terpene	Phosphonoglycans_biosynthetic_gene_cluster (3% of genes show similarity)	BGC0000806_c1
Cluster 10	Cf_putative	-	-
Cluster 11	Terpene	-	-
Cluster 12	Cf_putative	-	-
Cluster 13	Cf_putative	Azinomycin_B_biosynthetic_gene_cluster (4% of genes show similarity)	BGC0000960_c1
Cluster 14	Cf_putative	-	-
Cluster 15	Cf_saccharide	Polysaccharide_B_biosynthetic_gene_cluster (6% of genes show similarity)	BGC0001411_c1
Cluster 16	Cf_putative	Phosphonoglycans_biosynthetic_gene_cluster (6% of genes show similarity)	BGC0000807_c1
Cluster 17	Cf_putative	-	-
Cluster 18	Cf_putative	-	-
Cluster 19	T3pks	Alkyl-O-Dihydrogeranyl-Methoxyhydroquinones_biosynthetic_gene (71% of genes show similarity)	BGC0001077_c1
Cluster 20	Cf_saccharide	Coumermycin_A1_biosynthetic_gene_cluster (12% of genes show similarity)	BGC0000833_c1
Cluster 21	Cf_fatty_acid-Lantipeptide-	Kedarcidin_biosynthetic_gene_cluster (1% of genes show similarity)	BGC0000081_c1
Cluster 22	Cf_fatty_acid	Chlorizidine_A_biosynthetic_gene_cluster (7% of genes show similarity)	BGC0001172_c1
Cluster 23	Siderophore	Desferrioxamine_B_biosynthetic_gene_cluster (83% of genes show similarity)	BGC0000940_c1
Cluster 24	Cf_putative	-	-
Cluster 25	Cf_putative	Lobosamide_biosynthetic_gene_cluster (6% of genes show similarity)	BGC0001303_c1
Cluster 26	Cf_putative	Kanamycin_biosynthetic_gene_cluster (1% of genes show similarity)	BGC0000703_c1
Cluster 27	Cf_putative	-	-
Cluster 28	Lantipeptide	SRO15-3108_biosynthetic_gene_cluster (50% of genes show similarity)	BGC0000554_c1
Cluster 29	Cf_putative	-	-
Cluster 30	Cf_putative	Kanamycin_biosynthetic_gene_cluster (15% of genes show similarity)	BGC0000706_c1
Cluster 31	Cf_putative	-	-
Cluster 32	Cf_putative	Aclacinomycin_biosynthetic_gene_cluster (8% of genes show similarity)	BGC0000191_c1
Cluster 33	Cf_putative	-	-
Cluster 34	Cf_saccharide	-	-
Cluster 35	Cf_putative	-	-
Cluster 36	Cf_putative	Fengycin_biosynthetic_gene_cluster (20% of genes show similarity)	BGC0001095_c1
Cluster 37	Cf_saccharide	Nocathiacin_biosynthetic_gene_cluster (4% of genes show similarity)	BGC0000609_c1
Cluster 38	Nrps	Azicemicin_biosynthetic_gene_cluster (4% of genes show similarity)	BGC0000202_c1
Cluster 39	T2pks-Cf_fatty_acid	Pradimicin_biosynthetic_gene_cluster (25% of genes show similarity)	BGC0000256_c1
Cluster 40	Cf_putative	Taromycin_biosynthetic_gene_cluster (6% of genes show similarity)	BGC0000439_c1
Cluster 41	Cf_saccharide-Siderophore	Aldgamycin_biosynthetic_gene_cluster (5% of genes show similarity)	BGC0001396_c1
Cluster 42	Cf_putative	-	-
Cluster 43	Cf_putative	Lividomycin_biosynthetic_gene_cluster (10% of genes show similarity)	BGC0000708_c1
Cluster 44	Cf_putative	-	-
Cluster 45	Cf_putative	-	-
Cluster 46	Cf_putative	-	-
Cluster 47	Cf_putative	-	-
Cluster 48	Cf_putative	-	-
Cluster 49	Cf_putative	-	-
Cluster 50	Nrps	-	-
Cluster 51	T2pks-Siderophore	Xantholipin_biosynthetic_gene_cluster (20% of genes show similarity)	BGC0000279_c1
Cluster 52	Cf_putative	-	-
Cluster 53	Terpene	Meridamycin_biosynthetic_gene_cluster (13% of genes show similarity)	BGC0001011_c1
Cluster 54	Cf_putative	Svaricin_biosynthetic_gene_cluster (6% of genes show similarity)	BGC0001382_c1

***Micromonospora* sp. LB39**

Cluster	Type	Most similar known cluster	MIBiG BGC-ID
Cluster 1	Cf_putative	-	-
Cluster 2	Cf_putative	Svaricin_biosynthetic_gene_cluster (6% of genes show similarity)	BGC0001382_c1
Cluster 3	Cf_putative	Kanamycin_biosynthetic_gene_cluster (15% of genes show similarity)	BGC0000706_c1
Cluster 4	Cf_putative	-	-
Cluster 5	Cf_saccharide	Coumermycin_A1_biosynthetic_gene_cluster (12% of genes show similarity)	BGC0000833_c1
Cluster 6	Cf_saccharide	-	-
Cluster 7	Lassopeptide-Nrps	-	-
Cluster 8	Cf_putative	Kirromycin_biosynthetic_gene_cluster (3% of genes show similarity)	BGC0001070_c1
Cluster 9	Cf_putative	-	-
Cluster 10	Nrps	-	-
Cluster 11	Cf_fatty_acid	Alnumycin_biosynthetic_gene_cluster (6% of genes show similarity)	BGC0000195_c1
Cluster 12	Cf_putative	-	-
Cluster 13	T3pks	Alkyl-O-Dihydrogeranyl-Methoxyhydroquinones_biosynthetic_gene (71% of genes show similarity)	BGC0001077_c1
Cluster 14	Lantipeptide	-	-

Cluster 15	Cf_putative	-	-
Cluster 16	T2pks	Pradimicin_biosynthetic_gene_cluster (25% of genes show similarity)	BGC0000256_c1
Cluster 17	Cf_putative	Friulimicin_biosynthetic_gene_cluster (6% of genes show similarity)	BGC0000354_c1
Cluster 18	Cf_putative	-	-
Cluster 19	Cf_putative	-	-
Cluster 20	Cf_putative	-	-
Cluster 21	Cf_putative	-	-
Cluster 22	Cf_putative	Calcium-dependent_antibiotic_biosynthetic_gene_cluster (5% of genes show similarity)	BGC0000315_c1
Cluster 23	Cf_putative	-	-
Cluster 24	Cf_putative	Calicheamicin_biosynthetic_gene_cluster (2% of genes show similarity)	BGC0000033_c1
Cluster 25	Cf_saccharide	Polysaccharide_B_biosynthetic_gene_cluster (6% of genes show similarity)	BGC0001411_c1
Cluster 26	Siderophore	-	-
Cluster 27	Cf_putative	-	-
Cluster 28	Thiopeptide	-	-
Cluster 29	Cf_putative	-	-
Cluster 30	Cf_putative	A40926_biosynthetic_gene_cluster (7% of genes show similarity)	BGC0000289_c1
Cluster 31	Cf_putative	WS9326_biosynthetic_gene_cluster (5% of genes show similarity)	BGC0001297_c1
Cluster 32	Cf_putative	Asukamycin_biosynthetic_gene_cluster (4% of genes show similarity)	BGC0000187_c1
Cluster 33	Cf_putative	-	-
Cluster 34	T3pks-T1pks-Nrps	Sporolide_biosynthetic_gene_cluster (44% of genes show similarity)	BGC0000150_c1
Cluster 35	Cf_putative	Cyclomarin_biosynthetic_gene_cluster (8% of genes show similarity)	BGC0000333_c1
Cluster 36	Bacteriocin	Lymphostin_biosynthetic_gene_cluster (33% of genes show similarity)	BGC0001006_c1
Cluster 37	Lantipeptide-Nrps	Herboxidiene_biosynthetic_gene_cluster (2% of genes show similarity)	BGC0001065_c1
Cluster 38	Cf_putative	-	-
Cluster 39	Cf_putative	Friulimicin_biosynthetic_gene_cluster (6% of genes show similarity)	BGC0000354_c1
Cluster 40	Terpene	Phosphonoglycans_biosynthetic_gene_cluster (3% of genes show similarity)	BGC0000806_c1
Cluster 41	Cf_putative	-	-
Cluster 42	Cf_putative	-	-
Cluster 43	Cf_putative	-	-
Cluster 44	Cf_saccharide	Azinomycin_B_biosynthetic_gene_cluster (4% of genes show similarity)	BGC0000960_c1
Cluster 45	Cf_putative	-	-
Cluster 46	T2pks	Kinamycin_biosynthetic_gene_cluster (34% of genes show similarity)	BGC0000236_c1
Cluster 47	Terpene	-	-
Cluster 48	Cf_putative	-	-
Cluster 49	Cf_saccharide	Kinamycin_biosynthetic_gene_cluster (8% of genes show similarity)	BGC0000236_c1
Cluster 50	Cf_saccharide-Siderophore	-	-
Cluster 51	Terpene	Sioxanthin_biosynthetic_gene_cluster (40% of genes show similarity)	BGC0001087_c4
Cluster 52	Lantipeptide	SapB_biosynthetic_gene_cluster (100% of genes show similarity)	BGC0000551_c1
Cluster 53	Cf_putative	-	-
Cluster 54	Cf_saccharide	-	-
Cluster 55	Cf_putative	Pyrrolomycin_biosynthetic_gene_cluster (5% of genes show similarity)	BGC0000130_c1
Cluster 56	Cf_putative	-	-
Cluster 57	Cf_putative	-	-
Cluster 58	Cf_putative	Fengycin_biosynthetic_gene_cluster (13% of genes show similarity)	BGC0001095_c1
Cluster 59	Linaridin	Legonaridin_biosynthetic_gene_cluster (33% of genes show similarity)	BGC0001188_c1
Cluster 60	Cf_saccharide	Esmeraldin_biosynthetic_gene_cluster (8% of genes show similarity)	BGC0000935_c1
Cluster 61	Cf_putative	-	-
Cluster 62	Cf_putative	-	-
Cluster 63	Cf_putative	-	-
Cluster 64	T1pks-Nrps	Microsclerodermins_biosynthetic_gene_cluster (14% of genes show similarity)	BGC0001231_c1

***Micromonospora* sp. LB41**

**Cluster**	**Type**	**Most similar known cluster**	
Cluster 1	Cf_putative	-	-
Cluster 2	Lantipeptide	-	-
Cluster 3	Terpene	-	-
Cluster 4	Cf_saccharide	-	-
Cluster 5	Cf_putative	Diazepinomicin_biosynthetic_gene_cluster (15% of genes show similarity)	BGC0000679_c1
Cluster 6	Cf_putative	Friulimicin_biosynthetic_gene_cluster (6% of genes show similarity)	BGC0000354_c1
Cluster 7	T2pks	Xantholipin_biosynthetic_gene_cluster (16% of genes show similarity)	BGC0000279_c1
Cluster 8	Cf_fatty_acid	Chlorizidine_A_biosynthetic_gene_cluster (7% of genes show similarity)	BGC0001172_c1
Cluster 9	T1pks	Monensin_biosynthetic_gene_cluster (23% of genes show similarity)	BGC0000100_c1
Cluster 10	Cf_putative	-	-
Cluster 11	T1pks	Rubradirin_biosynthetic_gene_cluster (6% of genes show similarity)	BGC0000141_c1
Cluster 12	Cf_putative	-	-
Cluster 13	Lantipeptide	-	-
Cluster 14	Thiopeptide-Cf_saccharide	-	-
Cluster 15	T1pks-Nrps	Calicheamicin_biosynthetic_gene_cluster (22% of genes show similarity)	BGC0000033_c1
Cluster 16	T1pks	Nanchangmycin_biosynthetic_gene_cluster (30% of genes show similarity)	BGC0000105_c1
Cluster 17	Terpene	Phosphonoglycans_biosynthetic_gene_cluster (3% of genes show similarity)	BGC0000806_c1
Cluster 18	Cf_putative	-	-
Cluster 19	Cf_saccharide	-	-
Cluster 20	Nrps-T1pks-Otherks	Naphthyridinomycin_biosynthetic_gene_cluster (14% of genes show similarity)	BGC0000394_c1
Cluster 21	Cf_putative	-	-
Cluster 22	Cf_saccharide	Phosphonoglycans_biosynthetic_gene_cluster (14% of genes show similarity)	BGC0000806_c1
Cluster 23	Cf_putative	-	-
Cluster 24	Cf_putative	-	-
Cluster 25	Cf_putative	Meridamycin_biosynthetic_gene_cluster (5% of genes show similarity)	BGC0001011_c1
Cluster 26	Cf_putative	Polyoxypeptin_biosynthetic_gene_cluster (8% of genes show similarity)	BGC0001036_c1
Cluster 27	Cf_saccharide	Phosphonoglycans_biosynthetic_gene_cluster (6% of genes show similarity)	BGC0000807_c1
Cluster 28	Bacteriocin-Terpene	Lymphostin_biosynthetic_gene_cluster (30% of genes show similarity)	BGC0001007_c1
Cluster 29	Cf_putative	-	-
Cluster 30	Cf_putative	-	-
Cluster 31	Cf_fatty_acid-Nrps	Diazepinomicin_biosynthetic_gene_cluster (77% of genes show similarity)	BGC0000679_c1
Cluster 32	Oligosaccharide-Cf_fatty_a	Lobosamide_biosynthetic_gene_cluster (13% of genes show similarity)	BGC0001303_c1
Cluster 33	Cf_saccharide	-	-
Cluster 34	Cf_putative	Kanamycin_biosynthetic_gene_cluster (5% of genes show similarity)	BGC0000705_c1

Cluster 35	Cf_putative	-	-
Cluster 36	Siderophore-Nrps	Azicemicin_biosynthetic_gene_cluster (13% of genes show similarity)	BGC0000202_c1
Cluster 37	Cf_putative	Lasalocid_biosynthetic_gene_cluster (3% of genes show similarity)	BGC0000087_c1
Cluster 38	Cf_putative	Kanamycin_biosynthetic_gene_cluster (15% of genes show similarity)	BGC0000706_c1
Cluster 39	Cf_putative	-	-
Cluster 40	T1pks-Nrps	Phenalamide_biosynthetic_gene_cluster (33% of genes show similarity)	BGC0001394_c1
Cluster 41	T1pks	Incednine_biosynthetic_gene_cluster (6% of genes show similarity)	BGC0000078_c1
Cluster 42	Cf_putative	-	-
Cluster 43	Cf_putative	Pyrrolomycin_biosynthetic_gene_cluster (18% of genes show similarity)	BGC0000132_c1
Cluster 44	Cf_putative	-	-
Cluster 45	T1pks-Nrps	Bleomycin_biosynthetic_gene_cluster (12% of genes show similarity)	BGC0000963_c1
Cluster 46	Cf_putative	-	-
Cluster 47	Cf_putative	-	-
Cluster 48	Cf_putative	Coumermycin_A1_biosynthetic_gene_cluster (12% of genes show similarity)	BGC0000833_c1
Cluster 49	Cf_putative	Echosides_biosynthetic_gene_cluster (11% of genes show similarity)	BGC0000340_c1
Cluster 50	Terpene	Nocathiacin_biosynthetic_gene_cluster (4% of genes show similarity)	BGC0000609_c1
Cluster 51	Cf_putative	-	-
Cluster 52	Cf_putative	-	-
Cluster 53	Cf_putative	Rifamycin_biosynthetic_gene_cluster (15% of genes show similarity)	BGC0000137_c1
Cluster 54	T3pks	Alkyl-O-Dihydrogeranyl-Methoxyhydroquinones_biosynthetic_gene (71% of genes show similarity)	BGC0001077_c1
Cluster 55	Cf_putative	-	-
Cluster 56	Cf_putative	-	-
Cluster 57	Cf_putative	-	-
Cluster 58	Terpene	Sioxanthin_biosynthetic_gene_cluster (100% of genes show similarity)	BGC0001087_c4
Cluster 59	Cf_putative	-	-
Cluster 60	Cf_fatty_acid	Avilamycin_A_biosynthetic_gene_cluster (5% of genes show similarity)	BGC0000026_c1
Cluster 61	Cf_putative	Spinosad_biosynthetic_gene_cluster (8% of genes show similarity)	BGC0000148_c1
Cluster 62	Other	-	-
Cluster 63	Cf_putative	-	

In blue: differential cluster with previously described in *Micromonospora*[16]

Table S3. Number of genes of *Micromonospora* isolates implicated in DNA repair and stress responses found in the genomes.

		LB4	LB19	LB32^T	LB39^T	LB41
**DNA repair**						
alk B	Alkylated DNA repair protein	1	1	1	1	1
dcm	DNA-cytosine methyltransferase (EC 2.1.1.37)	1	0	0	1	1
din J	DNA damage inducible protein	0	0	1	1	0
din P	DNA polymerase IV (EC 2.7.7.7)	2	2	2	1	2
end 1	Endonuclease III (EC 4.2.99.18)	0	1	1	1	1
EndoIV	Endonuclease IV (EC 4.2.21.2)	2	2	2	2	2
EndoV	Endonuclease V (EC 4.2.21.7)	1	1	1	1	1
ExoIII	Exodeoxyribonuclease III (EC 3.1.11.2)	1	1	1	2	1
gly 1	Formamidopyrimidine-DNA glycosylase (EC 3.2.2.23)	3	4	4	3	4
gly 2	Uracil-DNA glycosylase, family 1	1	1	1	1	1
gly 3	DNA-3-methyladenine glycosylase (EC 3.2.2.20)	1	1	1	1	1
gly 4	DNA-3-methyladenine glycosylase II (EC 3.2.2.21)	0	1	1	1	1
Ku	Ku domain protein	1	1	1	1	1
lex A	SOS-response represor and protease (EC 3,4,21,88)	1	1	1	1	1
lig 1	ATP-dependent DNA ligase (EC 6.5.1.1)	3	5	5	5	4
lig 2	DNA ligase (EC 6.5.1.2)	1	1	1	2	1
lig C	ATP-dependent DNA ligase (EC 6.5.1.1)	1	2	1	3	1
lig D	ATP-dependent DNA ligase (EC 6.5.1.1) clustered with Ku protein	3	2	2	2	3
mug	G:T/U mismatch-specific uracil/thymine DNA-glycosylase	1	2	2	2	1
mut Y	A/G-specific adenine glycosylase (EC 3.2.2.-)	0	1	0	1	0
ogt	Methylated-DNA-protein-cystein methyltransferase (EC 2.1.163)	2	3	3	3	3
pol 1	DNA polymerase I (EC 2.7.7.7)	2	3	3	4	3
rad A	DNA repair protein	1	1	2	1	1
rec A	DNA repair protein	1	1	1	1	1
rec F	DNA recombination and repair protein RecF	1	1	1	1	1
rec N	DNA repair protein	1	1	1	1	1
rec O	DNA recombination and repair protein RecO	1	1	1	1	1
rec Q	ATP-dependent DNA helicase RecQ	3	3	3	4	4
rec R	Recombination protein RecR	1	1	1	1	1
rec X	Regulatory protein RecX	1	1	1	1	1
rmu C	DNA recombination protein	1	1	1	1	1
sbc C	Exonuclease	0	1	1	1	1
sbc D	Exonuclease	1	1	1	1	1
SCO5183	ATP-dependent DNA helicase SCO5183	1	1	1	1	1
SCO5184	ATP-dependent DNA helicase SCO5184	1	1	1	1	1
ssb	Single-stranded DNA-binding protein	2	3	2	3	2
UDG-1	Uracil-DNA glycosylase, family 1	1	1	1	1	1
UDG-2	G:T/U mismatch-specific uracil/thymine DNA-glycosylase	1	2	2	2	1
UDG-5	Uracil-DNA glycosylase, family 5	1	1	1	1	1
UDG-6	Uracil-DNA glycosylase, putative family 6	0	1	1	1	1
uvr A	Excinuclease ABC subunit A	1	2	1	1	1
uvr A-para	Excinuclease ABC subunit A paralog of unknown function	2	0	2	2	1
uvr B	Excinuclease ABC subunit B	1	1	1	1	1
uvr C	Excinuclease ABC subunit C	1	1	1	1	1
uvr D	ATP-dependent DNA helicase UvrD/PcrA	1	2	2	3	1
uvr D-actino	ATP-dependent DNA helicase UvrD/PcrA, actinomycete paralog	1	1	1	1	1
xse A	Exodeoxyribonuclease VII large subunit (EC 3.1.11.6)	1	1	1	1	1
xse B	Exodeoxyribonuclease VII small subunit (EC 3.1.11.6)	1	1	1	1	1
yoa A	DinG family ATP-dependent helicase YoaA	1	1	1	1	2
**Stress responses**						
-	CoA-disulfide reductase (EC 1.8.1.14)	1	1	1	1	1
1_Cl1	Copper binding protein, plastocyanin/azurin family	1	2	1	4	1

1_Cl2	Diguanylate cyclase/phosphodiesterase (GGDEF & EAL domains) with PAS/PAC sensor(s)	2	2	1	5	2
ahp C	Alkyl hydroperoxide reductase subunit C-like protein	3	4	4	4	3
aux	Cell wall endopeptidase, family M23/M37	0	0	0	0	1
aux	Similarity with glutathionylspermidine synthase (EC 6.3.1.8)	1	1	1	1	1
bet A	Choline dehydrogenase (EC 1.1.99.1)	0	0	0	1	1
bet C	Choline-sulfatase (EC 3.1.6.6)	1	1	2	2	1
chb	Cyanoglobin	1	1	1	1	2
csp A	Cold shock protein	4	5	4	3	4
csp C	Cold shock protein	1	1	1	1	1
ded A	DedA protein	2	4	4	4	2
dmaah	G,NG-dimethylarginine dimethylaminohydrolase 1 (EC 3.5.3.18)	2	2	2	2	2
dna J	Chaperone protein	2	3	2	4	2
dna K	Chaperone protein	1	2	1	4	2
Fe_stress	Phytochrome, two-component sensor histidine kinase (EC 2.7.3.-)	1	1	1	1	1
Fe^{3+}	ABC-type Fe^{3+}-siderophore transport system, permease 2 comp.	0	3	1	1	1
fur/zur	Transcriptional regulator, FUR family	3	3	3	3	3
gapdh	NAD-dependent glyceraldehyde-3-phosphate dehydrogenase (EC 1.2.1.12)	1	1	1	2	1
glo A	Lactoylglutathione lyase (EC 4.4.1.5)	1	2	3	3	1
glo B	Hydroxyacylglutathione hydrolase (EC 3.1.2.6)	1	1	1	1	1
grp E	Heat shock protein	1	1	1	1	1
GT	Glutathione S-transferase	1	1	1	1	1
hbo	Hemoglobin-like protein	1	2	1	2	1
hgl X	GTP-binding protein HflX	1	1	1	1	1
hmp X	Flavohemoprotein (Hemoglobin-like protein) (Flavohemoglobin) (Nitric oxide dioxygenase) (EC 1.14.12.17)	2	2	1	3	2
hpI	Peroxidase (EC 1.11.1.7)	1	1	1	1	1
hpII	Catalase (EC 1.11.1.6)	3	3	3	2	2
hrc A	Heat-inducible transcription repressor	1	1	1	1	1
hsp R	HspR, transcriptional repressor of DnaK operon	2	2	2	1	2
hyp1	Uncharacterized protein Rv0487/MT0505	0	1	1	0	1
hyp2	Putative hydrolase in cluster with formaldehyde/S-nitrosomycothiol reductase	0	2	1	0	1
lep A	Translation elongation factor	1	1	1	1	1
Mca	Mycothiol S-conjugate amidase	0	1	1	0	1
MPI	Maleylpyruvate isomerase, mycothiol-dependent (EC 5.2.1.4)	0	1	1	0	1
msc R1	S-nitrosomycothiol reductase	0	1	1	0	1
msc R2	Formaldehyde dehydrogenase MscR, NAD/mycothiol-dependent (EC 1.2.1.66)	0	1	1	0	1
msh A	Glycosyltransferase MshA involved in mycothiol biosynthesis (EC 2.4.1.-)	0	1	2	0	1
msh B	N-acetyl-1-D-myo-inositYl-2-amino-2-deoxy-alpha-D-glucopyranoside deacetylase	0	1	1	0	1
msh C	L-cysteine:1D-myo-inositYl 2-amino-2-deoxy-alpha-D-glucopyranoside ligase	0	1	1	0	1
msh D	Acetyl-CoA:Cys-GlcN-Ins acetyltransferase, mycothiol synthase	0	1	1	0	2
mtr	NADPH-dependent mycothiol reductase	0	1	1	0	1
npt 1	Nicotinate phosphoribosyltransferase (EC 2.4.2.11)	1	1	1	1	1
nsr R	Nitrite sensitive transcriptional represor	0	0	0	1	2
oat	Ornithine aminotransferase (EC 2.6.1.13)	1	1	1	1	1
omp A	Aquaporin Z	0	1	1	1	0
opu A	Glycine betaine ABC transport system	0	0	0	1	0
opu D	Glycine betaine transporter	0	0	0	0	1
orgH2O2	Organic hydroperoxide resistance protein	5	4	4	5	5
osm Y	Osmotically inducible protein Y	0	1	1	1	0
pnc1	Nicotinamidase (EC 3.5.1.19)	1	1	1	1	1
prmt12	Protein arginine N-methyltransferase 1 (EC 2.1.1.-)	1	0	0	0	1
pro U	Glycine betaine ABC transport system protein	2	4	4	9	3
prxcat	Catalase (EC 1.11.1.6) EC Number 1.11.1.6 / Peroxidase (EC 1.11.1.7)	3	4	4	4	3
psp	Putative SigmaB asociated two-component system sensor protein	2	2	2	2	2
qor 2	NADPH: quinone oxidoreductase 2	0	2	1	2	0
qor R	Redox-sensing transcriptional regulator	0	1	1	1	0
qor R_p	Redox-sensing transcriptional regulator putative	0	1	0	0	0
rdg B	Nucleoside 5-triphosphatase RdgB (dHAPTP, dITP, XTP-specific) (EC 3.6.1.15)	1	1	1	1	1

redox	Redox-sensitive transcriptional regulator (AT-rich DNA-binding protein)	1	1	1	1	1
RNAse	Ribonuclease PH (EC 2.7.7.56)	1	1	1	1	1
rsb R	Positive regulator of Sigma-B	1	1	1	1	1
rsb S	Negative regulator of Sigma-B	1	1	1	1	1
rsb U	Serine phosphatase, regulator of sigma unit	8	9	11	8	9
rsb V	Anti-Sigma B factor antagonist	3	2	4	2	3
rsb W	Serine-protein kinase (EC 2.7.11.1)	2	2	2	2	2
rsm E	Ribosomal RNA small subunit methyltransferase E (EC 2.1.1.-)	1	1	1	1	1
rsm I	rRNA small subunit methyltransferase I	0	1	1	1	1
rsp A	Starvation sensing protein	1	1	1	1	1
sam	Hypothetical radical SAM family enzyme in heat shock gene cluster, similarity with CPO of BS HemN-type	0	1	2	2	1
sig B	RNA polymerase sigma factor	5	5	3	3	5
sir2	NAD-dependent protein deacetylase of SIR2 family	1	1	1	1	2
smo F	Various polyols ABC transporter, permease component 1	0	1	2	1	0
smp B	tmRNA-binding protein SmpB	2	2	2	2	2
sod B	Superoxide dismutase [Fe] (EC 1.15.1.1)	2	1	0	0	2
sox R	Redox-sensitive transcriptional activator	2	1	3	1	2
sp	Signal peptidase-like protein	1	1	1	1	1
temp	protein similar to cysteinyl-tRNA synthetase and MshC	0	1	1	0	1
zur	Zinc uptake regulation protein	1	1	1	1	1

Figure S1. RAPD profiles showing genetic variability between isolates LB4, LB19, LB32^{T}, LB39^{T} and LB 41.

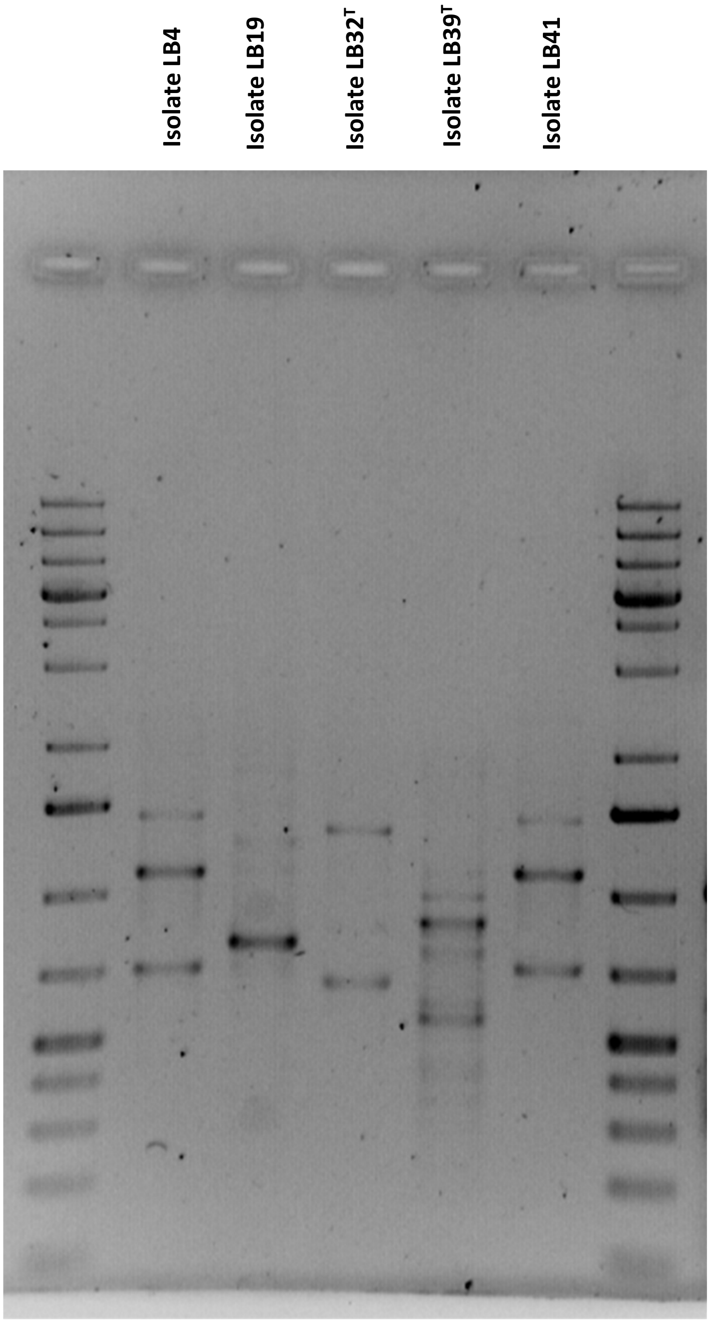

Figure S2. Neighbour-joining phylogenetic tree based on 16S rRNA gene sequences showing relationships between the isolates and between them and *Micromonospora* type strains. The numbers at the nodes indicate bootstrap values > 50%. Asterisks indicate branches of the tree that were also recovered in the maximum-likelihood tree. Bar, 0.005 substitutions per nucleotide position.

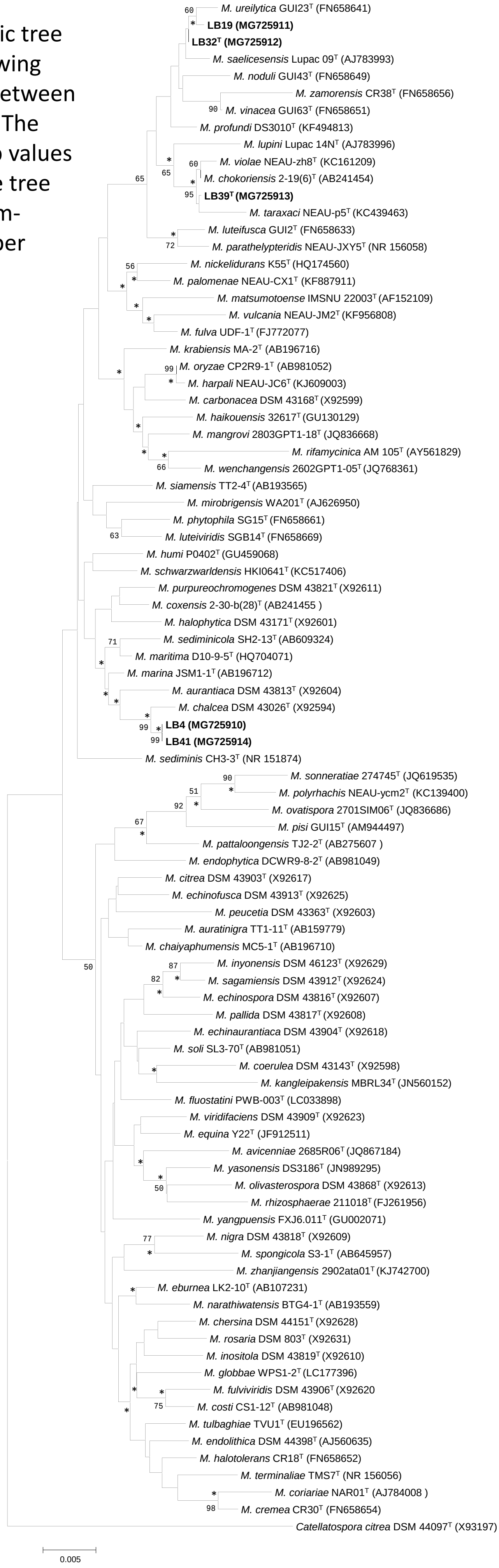

Supplement: Supplementary file 1 — Supplementary data [file 41598_2019_38789_MOESM1_ESM.pdf]
